# Multi-Gram Scale Synthesis and Characterization of Mometasone Furoate EP Impurity C

**DOI:** 10.3390/molecules28237859

**Published:** 2023-11-30

**Authors:** Riccardo Ronchetti, Luigi Alfonso Pannone, Bruno Cerra, Emidio Camaioni, Gianfranco Lopopolo, Emanuele Attolino, Antimo Gioiello

**Affiliations:** 1 Laboratory of Medicinal and Advanced Synthetic Chemistry (Lab MASC), Department of Pharmaceutical Sciences, University of Perugia, Via del Liceo 1, 06122 Perugia, Italy; riccardo.ronchetti@chimfarm.unipg.it (R.R.); luigialfonso.pannone@studenti.unipg.it (L.A.P.); emidio.camaioni@unipg.it (E.C.); antimo.gioiello@unipg.it (A.G.); 2 Research & Development Department, Newchem SpA, Via Roveggia, 47, 37136 Verona, Italy; gianfranco.lopopolo@newchemspa.it (G.L.); emanuele.attolino@newchemspa.it (E.A.)

**Keywords:** mometasone furoate, mometasone furoate EP impurity C, synthesis of APIs impurities, steroid

## Abstract

Mometasone furoate is a synthetic corticosteroid used in the treatment of skin inflammatory conditions, hay fever and asthma. The industrial manufacturing routes to mometasone furoate are generally accompanied by the formation of numerous process impurities that need to be detected and quantified, as requested by regulatory authorities. The ready availability of such impurities in the required quantity and purity is therefore essential for toxicological studies, analytical method development and process validation. Herein, we report the multi-gram scale preparation of 21′-chloro-(16′α-methyl-3′,11′,20′-trioxo-pregna-1′,4′-dien-17′-yl)-furan-2-carboxylate (mometasone furoate EP impurity C), one of the known impurities of mometasone furoate. This study also includes the systematic investigation of the final acylation step, as well as the characterization of the difuroate enol ether intermediate and its conversion to the target impurity C.

## 1. Introduction

**Scheme 1:**
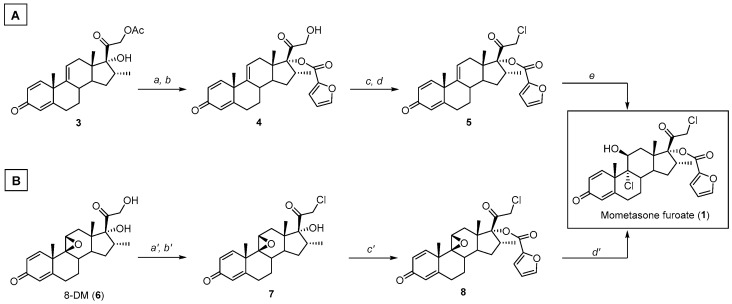
Synthetic approaches reported in the literature for the preparation of mometasone furoate (MF, **1**) [4,8,9]. (**A**) Reagents and conditions: (a) 2-furoyl chloride, DMAP, CH_2_Cl_2_, 25 °C; (b) HClO_4_, MeOH, 25 °C; (c) MsCl, pyridine, 0 °C; (d) LiCl, DMF, 80 °C; (e) 1,3-Dichloro-5,5-dimethylhydantoin, HClO_4_, H_2_O, THF, 0 °C, then 25 °C, 68% overall yield from **3**. (**B**) Reagents and conditions*:* (a’) TsCl, Et_3_N, CH_2_Cl_2_, 0 °C; (b’) heating (40 °C); (c’) 2-Furoyl chloride, Et_3_N, −5 °C; (d’) HCl, AcOH, 0 °C, 80% overall yield from **6**.

Mometasone furoate (MF, **1**, Scheme 1) is a steroidal active pharmaceutical ingredient (API) employed as ointment, cream or lotion in the treatment of inflammatory skin disorders such as atopic dermatitis eczema and psoriasis [1]. MF (**1**) is also used as metered spray to alleviate the symptoms of seasonal allergic rhinitis (e.g., hay fever) and to treat nasal polyps [2], as well as in aerosol and dry powder inhalers for the prevention of asthma attacks [3]. MF (**1**) was patented by Schering Corp. in 1981 and launched on the market by Merck Sharp & Dohme Corp./Schering Corp. in 1987 under the brand name of Elocon^®^ [4,5]. MF is mentioned on the World Health Organization’s list of essential medicines and among the top 300 most prescribed drugs in the United States in 2020 [6,7]. The processing methods of MF (**1**) are based on two approaches that share the same reaction steps but in a different order and starting material (Scheme 1) [4,8,9]. Although some minor changes have been proposed over the years to improve both the product yield and quality profile [10], the process to MF (**1**) generally leads to the formation of numerous impurities [11]. According to the International Conference on Harmonization (ICH) guidelines, impurity profiling represents a mandatory duty within the production process of APIs and involves both toxicological assessments and analytical activities aimed at detecting, elucidating and quantifying the API-related, process-related or stability-related impurities [12,13,14,15,16,17]. Moreover, impurity profiling plays a crucial role in the routine activities of modern API manufacturing including in-process control, method and process validation, definition of starting materials/reagents specifications and carry-over activities. In addition, the impurity profiling requires intensive efforts for drawing ad hoc synthetic routes of high-purity reference standards for impurities that are present at a very low level (e.g., 0.10%) in the final API and, therefore, are difficult to isolate [18]. Pharmaceutical companies frequently outsource to contract development and manufacturing organizations (CDMOs) the management of impurity synthesis for supporting regulatory requirements with a subsequent increase in the cost for process development studies.

Twenty known impurities, namely impurities A-T, and one unknown impurity (impurity U) are reported in the corresponding monograph of MF (**1**) in the European *Pharmacopoeia* [11]. Although known MF impurities are commercially available from specialized suppliers [19], the procedures for the preparation of most of them are not available in the literature. Recently, Das and co-workers reported the preparation of some impurities of corticosteroids starting from tetraene acetate (**3**) and, among them, the synthesis of six impurities related to MF (impurities G, H, K, L, N and Q) [20]. Although structurally similar to each other and related to the structure of MF (**1**), the preparation of these impurities requires the design and the development of ad hoc synthetic processes to provide the target impurity on-demand, in the desired amount and purity grade.

**Scheme 2:**
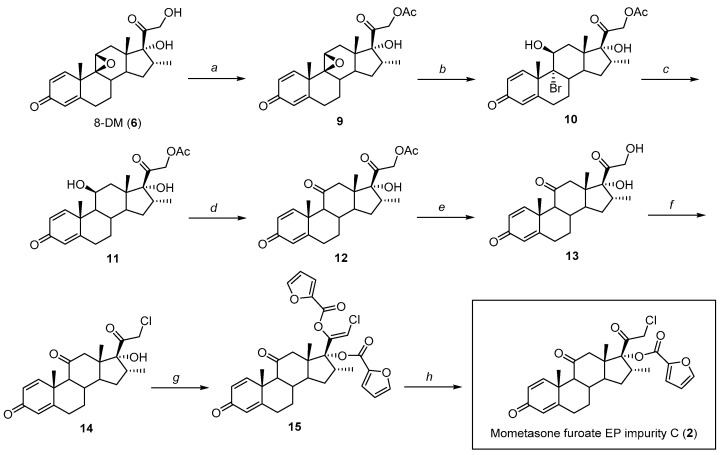
Synthesis of 21′-chloro-(16′α-methyl-3′,11′,20′-trioxo-pregna-1′,4′-dien-17′-yl)-furan-2-carboxylate (mometasone furoate EP impurity C, **2**). Reagents and conditions: (a) Ac_2_O, KOAc, *N,N*-dimethylacetamide (DMAC), 25 °C; (b) HBr (48% aq.), AcOH, 0 °C, then 25 °C; (c) CrCl_3_ . 6H_2_O, Zn dust, *N,N*-dimethylformamide (DMF), dimethyl sulfoxide (DMSO), thioglycolic acid, 0 °C; (d) Pyridinium chlorochromate (PCC), CH_2_Cl_2_, 0 °C, then 25 °C, 71% isolated yield (crystallization) from **6**; (e) NaHCO_3_, MeOH, 80 °C; (f) 4-Dimethylaminopyridine (DMAP), methanesulfonyl chloride (MsCl), CH_2_Cl_2_, 0 °C, then reflux; (g) Et_3_N, DMAP, 2-furoyl chloride, CH_2_Cl_2_, 0 °C, then 25 °C, 57% isolated yield (silica filtration) from **12**; (h) HClO_4_, CH_2_Cl_2_, −10 °C, 73% isolated yield (chromatography and crystallization) from **15**, 30% overall yield from **6**, ≥96% purity.

In this context, herein we report the synthesis of 21′-chloro-(16′α-methyl-3′,11′,20′-trioxo-pregna-1′,4′-dien-17′-yl)-furan-2-carboxylate (mometasone furoate EP impurity C, **2**) (Scheme 2). This impurity (CAS: 1305334-31-9) is commercially available as a reference standard (mg scale) from different specialized suppliers [21], but its preparation has never been reported so far, thus making the development of a reliable synthetic route for its preparation highly desirable for the steroidal community.

## 2. Results and Discussion

The synthesis of the target product **2** started from the cheap and readily available 9β,11β-epoxy-17α,21-dihydroxy-16α-methyl-3,20-dioxo-pregna-1,4-diene (**6**), namely 8-DM (Scheme 2) [22]. Being **2** a process impurity structurally related to the parent API, we adopted a strategy similar to the synthetic sequence for the manufacturing of MF (**1**) shown in Scheme 1B. The synthesis consisted of the initial manipulation of ring C by converting the 11,12-epoxide moiety to the desired C11-oxo functionality, followed by the chlorination at C21-position and the final acylation reaction at the C17-position (Scheme 2). Initially, the route was attempted at mg scale. Thus, 8-DM (**6**) was treated with acetic anhydride and potassium acetate in *N,N*-dimethylacetamide (DMAC) at 25 °C affording the corresponding 21-acetoxy intermediate **9** (*step a*). The crude **9** obtained by aqueous extractive work-up (95% recovery) was then submitted to the epoxide ring-opening by treatment with hydrobromic acid in AcOH at 25 °C. After aqueous extractive work-up, the desired bromohydrin derivative **10** was isolated in 90% yield and good purity (*step b*). The crude bromohydrin **10** was then de-halogenated under Barton’s conditions [23,24] by treatment with CrCl_3_·6H_2_O, zinc dust and thioglycolic acid in DMF and DMSO affording the desired 11β-hydroxy intermediate **11** in 82% isolated yield from **6** after chromatographic purification (*step c*). Oxidation of **11** by treatment with pyridinium chlorochromate (PCC) in CH_2_Cl_2_ afforded the corresponding 11-oxo derivative **12** in 74% yield after chromatographic purification (*step d*). Deprotection of the acetyl group at C21-position was achieved under mild alkaline conditions with 95% crude recovery after aqueous extractive work-up (*step e*). The C21 free hydroxyl group was activated by treatment with methanesulfonyl chloride (MsCl) and 4-dimethylaminopyridine (DMAP) in CH_2_Cl_2_ at 0 °C. The methanesulfonate intermediate thus obtained was converted into the corresponding 21-chloro derivative **14** by heating the crude mixture (Scheme 2, *step f*). The crude **14** was finally submitted to the furoylation reaction at the C-17 tertiary alcohol group (*step g*).

The reaction was initially performed using pyridine (2 equiv.) as the base and 2-furoyl chloride (1.2 equiv.) in CH_2_Cl_2_ at 25 °C. Under these conditions, a partial conversion of **14** was observed affording the target product **2** in 27% isolated yield (Table 1, *entry 1*). The use of Et_3_N (2 equiv.) instead of pyridine in the presence of DMAP (0.1 equiv.) as the acyl transfer reagent resulted in the formation of the target product **2** (46% yield) along with unreacted **14** after 24 h at 25 °C (*entry 2*). However, when the reaction was scaled-up at 1 g, a longer reaction time (up to 48 h) and higher temperature (reflux) were needed (*entry 3*). Interestingly, the use of higher amounts of Et_3_N (4 equiv.), DMAP (0.25 equiv.) and 2-furoyl chloride (2 equiv.) resulted in the almost quantitative consumption of **14** and in the formation of a different reaction product (76% yield) with a TLC retention factor similar to that of the target product **2** (Table 1, *entry 4*).

**Scheme 3:**
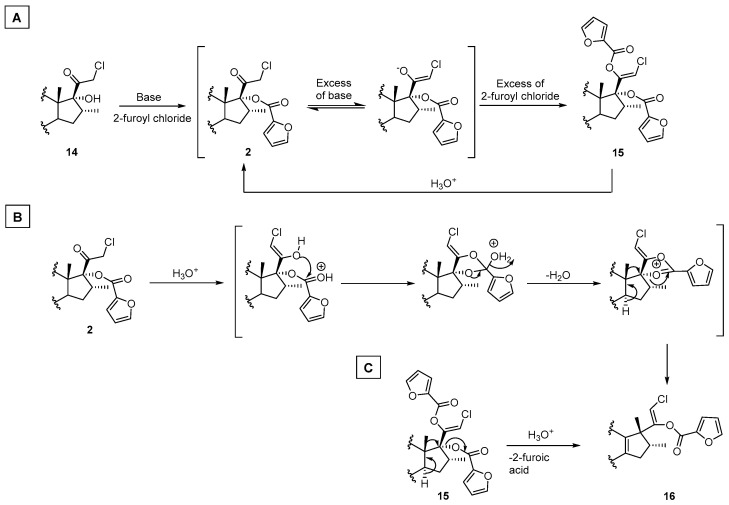
(**A**) Proposed mechanism for the formation and acid-promoted hydrolysis of difuroate enol ether **15** from intermediate **14**; (**B**) proposed mechanism for the formation of rearranged side product **16** by acid−catalysed migration of the angular 18−CH_3_ by stepwise pathway from target product **2**, or (**C**) by concerted mechanism from enol ether **15** [25].

High resolution mass spectrometry (HRMS) analysis showed a mass of *m*/*z* 579.1785 that corresponds to the formula C_32_H_31_ClO_8_ in positive ion mode. Nuclear magnetic resonance (NMR) analysis confirmed the presence of a second 2-furoyl group at the side chain. In particular, the comparison of the ^1^H- and ^13^C-NMR data with that of compound **2** clearly evidenced the loss of 21-methylene protons at around 4 ppm and the formation of a double bond associated with a single vinylic proton (singlet at 6.31 ppm and *C*H at 111.5 ppm) of the chloro vinyl ether moiety bearing the second furoyl group. Moreover, key spatial correlations in the NOESY experiment confirmed the *Z*-geometry at the double bond of the chloro vinyl ether group (see Supplementary Materials). Based on the analytical and spectroscopic data and supported by the literature analysis [25], we unambiguously assigned the structure of the side product as the difuroate enol ether **15**. It is worth noting that although **15** is not recognized among the known process impurities of MF (**1**), it is commercially available from some specialized suppliers of API impurities [26]. We therefore focused our efforts at finding mild hydrolytic conditions to convert difuroate enol ether **15** to the desired mometasone furoate EP impurity C (**2**) (Table 2). Heggie et al. reported the conversion of traces of difuroate side products like **15** to MF (**1**) by treatment with concentrated HCl and AcOH at 15–25 °C [10]. However, under these conditions **15** may undergo an acid-catalysed migration of the angular 18-CH_3_ group to the electron deficient C-17 carbon atom via a carbonium ion by either a stepwise or concerted mechanism (Scheme 3) [25].

In our case, the use of 12 N HCl resulted in the formation of unidentified side product(s) along with the almost quantitative consumption of **15** (Table 2, *entries 1–3*). Also, the use of 3 N HCl in AcOH at 0 °C and *p*-toluenesulfonic acid (*p*TSA) in H_2_O/acetone gave traces of the desired target product **2** along with unreacted **14** (*entries 4* and *5*). Interestingly, the use of HClO_4_ in CH_2_Cl_2_ at 0 °C afforded the target product **2** in 46% isolated yield (*entry 6*). Lower temperatures (−10 °C) and diluted conditions gave a better outcome (72% yield) (Table 2, *entry 7*).

Accordingly, the target product **2** was obtained in 33% yield from 8-DM (**6**) over eight steps at mg scale (Scheme 2 and Table 3). Having optimized the conversion of the difuroate enol ether **15** to the target mometasone furoate EP impurity C (**2**) (*step h*) and evaluated the feasibility of the synthetic strategy shown in Scheme 2, we finally aimed at scaling the process at the multi-gram scale (27 g of **6**). At this point, in order to make the synthetic route easily applicable in industrial settings, we focused on improving work-up and isolation procedures by avoiding, when possible, tedious chromatographic purifications and aqueous extractive work-ups (Table 3). In particular, intermediates **9** and **10** were obtained by simple precipitation from water, the chromatographic purification of **11** was replaced by crystallization and intermediate **15** was purified by simple filtration on silica gel. Flash chromatography followed by crystallization were required to obtain **2** in 30% overall yield and ≥96% purity at multi-gram scale with solvent-saving isolation and purification procedures with respect to initial small-scale synthesis (Table 3).

Target product **2** was fully characterized by mono- and bidimensional NMR, HRMS and IR analyses, while its purity (≥96%) was assessed by quantitative normal phase (NP) and reverse phase (RP) C18-thin layer chromatography, quantitative ^1^H-NMR in the presence of dimethyl sulfone (DMS) as the internal standard and high-performance liquid chromatography (HPLC) analysis (see Supplementary Materials). HRMS analysis in positive ion mode showed a *m*/*z* 485.1728, which corresponds to the formula C_27_H_29_ClO_6_, and a *m*/*z* 486.1759, which corresponds to the *p* + 1 (abund. 27.29%), conforming the presence of a chlorine atom. The comparison of ^1^H- and ^13^C-NMR spectra of **2** with those of the starting material 8-DM (**6**) confirmed the presence of the conjugated dienone at the A ring of the steroid backbone (signals at δ 6.11, 6.23 and 7.68 ppm in the proton spectra and carbon nuclei at δ 186.2, 165.9, 154.8, 127.8 and 124.3 ppm). The presence of two additional carbonyl groups at C11 and C20 position was proved by the signals at δ 207.5 and 196.1, respectively, in the carbon spectrum. The presence of a methylene group bearing the chlorine atom at the side chain was confirmed by the presence of a multiplet at 4.03–4.11 ppm, which corresponds to the signal at 44.6 ppm in the ^13^C-NMR spectrum. Finally, the signals at δ 6.57, 7.29 and 7.64 in the ^1^H-NMR spectrum and the carbon nuclei at δ 112.5, 120.2, 142.8, 147.5 and 158.0 ppm are related to the presence of the 2-furoyl group at the C17 position (carbon at 96.8 ppm). Scalar and spatial correlation in homonuclear ^1^H-^1^H COSY and NOESY experiments confirmed the aforementioned atom arrangement (see Supplementary Materials).

## 3. Material and Methods

### 3.1. General Methods

Unless otherwise noted, chemicals were obtained from commercial suppliers and used without further purification. Reactions requiring anhydrous conditions were conducted in dried glass apparatus under a positive pressure of N_2_, using freshly distilled solvent according to reported procedures. NMR spectra were recorded on Brucker Ascend^TM^ 600 MHz (superconducting magnet) or Bruker AC 400 MHz (Bruker, Madison, WI, USA). Chemical shifts are reported in parts per million (ppm) and are relative to CDCl_3_ (7.26 ppm and 77.0 ppm). The abbreviations used are as follows: s, singlet; brs, broad singlet; d, doublet; dd, double of doublets; dt, doublet of triplets; t, triplet; q, quartet; qui, quintet; m, multiplet; and brm, broad multiplet. Coupling constants (J) are reported in Hertz (Hz). Thin-layer chromatography (TLC) was performed on aluminium backed silica plates or RP-18 glass plates (silica gel 60 F254, Merck, Darmstadt, Germany). Spots were visualized by UV detector (λ: 254 nm) and/or by staining and warming with phosphomolybdic acid (5% *w*/*v* solution in EtOH). When required, flash chromatographic purifications were performed using Biotage Isolera (Biotage AB, Uppsala, Sweden). Uncorrected melting points were determined using a Stuart SMP3 apparatus (Bibby Scientific, Milan, Italy). IR spectra were recorded using an IRSpirit–Shimadzu FTIR/ATR apparatus (Shimadzu, Kyoto, Japan) in the spectral range of 400–5000 cm^−1^ and with a resolution of 4 cm^−1^ at 50 scans. Purity of the target product 2 was assessed by quantitative NP- and RP-18 TLC using 8-DM (6) as the reference standard, qNMR in the presence of dimethyl sulfone (standard for quantitative NMR, TraceCERT^®^, Merck KGaA, Darmstadt, Germany) as the internal standard (acquisition parameters: td: 65.536, ds: 0, ns: 2, sw: 19.8368, o1p: 6.175, o2p: 6.175. rg: 16) and by HPLC analysis. HPLC analyses (method Ph. Eur. 8.0 for mometasone furoate) were performed on a Shimadzu (Kyoto, Japan) LC-20A Prominence equipped with a CBM-20A communication bus module, two LC-20AD dual piston pumps, an SPD-M20A photodiode array detector, and a Rheodyne 7725i injector (Rheodyne Inc., Cotati, CA, USA) with a 20-μL stainless steel loop. A GraceSmart RP18 column (Grace, Sedriano, Italy, 250 × 4.6 mm i.d., 5 mm, 100 Å) was used as the analytical column. The analysis was performed at a 25 °C column temperature using analytical grade MeCN and ultrapure water (σ = 18.3 MΩ × cm) obtained from a Milli-Q Plus185 system from Millipore (Milford, MA, USA). The HPLC analysis was performed at 1.0 mL min^−1^ eluent flow rate for a running time of 100 min and at 254 nm of detection wavelength, after previous conditioning by passing through the column the selected mobile phase (H_2_O/MeCN, 50:50, *v*/*v* + 0.1% AcOH) for at least 30 min at the same eluent flow rate. Before use, the mobile phase was filtered through a 0.22 mm Millipore filter (Bedford, MA, USA) and then degassed with 20 min sonication. The column temperature was controlled through a heather/chiller thermostat (Grace 7956 R, Grace, Sedriano, Italy). Samples of the target product were prepared by dissolving 2 at 1 mg mL^−1^ in analytical grade MeCN and 20 μL were injected. High resolution mass spectrometry (HRMS) measurements were performed on a UHPLC–MS/MS system consisting of an Agilent 1290 Infinity II module combined with an Agilent 6560 mass spectrometer (Agilent Technologies Inc., Santa Clara, CA, USA). The chromatographic separation was performed using a Zorbax Rrhd Eclipse Plus C18 column (50 mm × 2.1 mm, 1.8 µm, 95 Å, Agilent Technologies Inc.). HPLC eluent A was water (LC-MS grade, LiChrosolv, Supelco, Merck KGaA, Darmstadt, Germany) with 0.1% (*v*/*v*) of formic acid (LC-MS grade, LiChropur, Supelco), while eluent B was MeCN (LC-MS grade, LiChrosolv, Supelco). The gradient was 50% B for 1 min, then a linear increase took place up to 97% B over 6 min and this condition was maintained for 5 min. Finally, the system returned to 50% B in 1 min and was re-equilibrated for 2 min. The column temperature was kept at 25 °C and the flow rate was 0.3 mL min^−1^. The injection volume was 5 µL. Samples were dissolved in MeOH (LC-MS grade, LiChrosolv, Supelco) and sonicated for 5 min. MassHunter software version B.05.01 (Agilent Technology) was used to control the LC–MS/MS system, for data prediction, acquisition, analysis and processing. For MS detection, the Dual AJS ESI source operated in positive ion mode. The gas temperature was set at 300 °C with a flow of 5 L min^−1^ while the sheath gas temperature was 350 °C with a flow of 11 L min^−1^. The nebulizer pressure was set at 35 psi, while the capillary and fragmentation voltages were 3500 V and 400 V, respectively.

### 3.2. Synthesis

#### 3.2.1. Synthesis of 9β,11β-Epoxy-17α-hydroxy-16α-methyl-3,20-dioxo-pregna-1,4-dien-21-acetate (**9**)

To a solution of dexamethasone 9,11-epoxide (8-DM, **6**) (27 g, 72.5 mmol) in *N,N*-dimethylacetamide (120 mL), AcOK (12.5 g, 127.5 mmol) and Ac_2_O (30 mL, 318.0 mmol) were sequentially added. The reaction mixture was stirred for 1 h at 25 °C. The reaction mixture was then poured into ice/H_2_O (600 mL) and kept under vigorous stirring for 30 min. The precipitate thus formed was collected by filtration under vacuum and washed with H_2_O (300 mL). The solid was dried in a vacuum oven (45 °C, 16 h, 100 mmHg) affording the desired 9β,11β-epoxy-17α-hydroxy-16α-methyl-3,20-dioxo-pregna-1,4-dien-21-acetate (**9**, 29.8 g, 71.8 mmol, 99% recovery, ≥97% purity by q^1^H-NMR) as a white solid (m.p.: 192–194 °C) that was used for the next step without further purification. ^1^H-NMR (400 MHz, CDCl_3_): δ 0.88 (d, *J* = 7.21 Hz, 3H, 16α-C*H_3_*), 0.93 (s, 3H, 18-C*H_3_*), 1.44 (s, 3H, 19-C*H_3_*), 2.16 (s, 3H, OCOC*H_3_*), 3.22 (s, 1H, 11α-C*H*), 4.62–5.05 (m, 2H, 21-C*H_2_*OCOCH_3_), 6.15 (s, 4-C*H*), 6.19 (dd, *J_1_* = 1.77 Hz, *J_2_* = 10.09 Hz, 1H, 2-C*H*), 6.60 (d, *J* = 10.08 Hz, 1H, 1-C*H*). ^13^C-NMR (100.6 MHz, CDCl_3_): δ 14.5, 17.3, 20.4, 23.7, 29.1, 29.9, 30.4, 33.2, 34.2, 35.3, 44.1, 47.6, 48.1, 62.7, 66.0, 67.5, 90.5, 125.0, 127.9, 152.2, 164.9, 170.6, 186.0, 204.6.

#### 3.2.2. Synthesis of 9α-Bromo-11β,17α-dihydroxy-16α-methyl-3,20-dioxo-pregna-1,4-dien-21-acetate (**10**)

To a solution of 9β,11β-epoxy-17α-hydroxy-16α-methyl-3,20-dioxopregna-1,4-dien-21-yl acetate (**9**, 29.7 g, 71.7 mmol) in AcOH (135 mL), 48% aqueous solution of HBr (8 mL) was added dropwise over 15 min at 0 °C. The resulting mixture was stirred at 0 °C for 30 min and then allowed to warm to 25 °C and stirred for a further 2 h. The reaction mixture was then poured into ice/H_2_O (600 mL) and kept under vigorous stirring for 30 min. The precipitate thus formed was collected by filtration under vacuum and washed with H_2_O (300 mL). The solid was dried in a vacuum oven (45 °C, 16 h, 100 mmHg) affording the desired 9α-bromo-11β,17α-dihydroxy-16α-methyl-3,20-dioxo-pregna-1,4-dien-21-acetate (**10**, 33.1 g, 66.7 mmol, 93% recovery, ≥90% purity by q^1^H-NMR) as a white solid that was used for the next step without further purification. ^1^H-NMR (400 MHz, CDCl_3_): δ 0.86 (d, *J* = 7.33 Hz, 3H, 16α-C*H*_3_), 0.97 (s, 3H, 18-C*H*_3_), 1.67 (s, 3H, 19-C*H*_3_), 2.14 (s, 3H, OCOC*H*_3_), 4.68–4.70 (m, 1H, 11α-C*H*), 4.86 (*pseudo*-q, *J* = 17.57, 2H, 21-C*H*_2_OCOCH_3_), 6.02 (s, 4-C*H*), 6.29 (dd, *J*_1_ = 1.87 Hz, *J_2_* = 10.08 Hz, 1H, 2-C*H*), 7.28 (d, *J* = 10.11 Hz, 1H, 1-C*H*). ^13^C-NMR (100.6 MHz, CDCl_3_ + CD_3_OD): δ 14.6, 17.0, 20.6, 24.9, 28.8, 30.6, 31.8, 35.3, 35.5, 35.8, 45.2, 48.7, 50.5, 68.9, 75.5, 85.9, 91.1, 124.5, 128.7, 154.0, 167.6, 171.7, 187.1, 205.1.

#### 3.2.3. Synthesis of 11β,17α-Dihydroxy-16α-methyl-3,20-dioxopregna-1,4-dien-21-acetate (**11**)

To a solution of 9α-bromo-11β,17α-dihydroxy-16α-methyl-3,20-dioxopregna-1,4-dien-21-yl acetate (**10**, 33.0 g, 66.6 mmol) in DMF (100 mL), DMSO (12 mL), CrCl_3_∙6H_2_O (2.48 g, 9.3 mmol), Zn dust (3.1 g, 46.6 mmol) and thioglycolic acid (8.3 mL, 118.7 mmol) were sequentially added at 0 °C. The resulting suspension was stirred at 0 °C for 4 h. The reaction mixture was then poured into ice/H_2_O (600 mL) and kept under vigorous stirring for 30 min. The precipitate thus formed was collected by filtration under vacuum and washed with H_2_O (300 mL). The solid was dried in a vacuum oven (45 °C, 16 h, 100 mmHg) affording the desired 11β,17α-dihydroxy-16α-methyl-3,20-dioxopregna-1,4-dien-21-acetate (**11**, 27.5 g, 66.0 mmol, 99% recovery, ≥87% purity by q^1^H-NMR) as a greenish solid that was used for the next step without further purification. ^1^H-NMR (400 MHz, CDCl_3_): δ 0.90 (d, *J* = 7.29 Hz, 3H, 16α-C*H_3_*), 1.06 (s, 3H, 18-C*H_3_*), 1.44 (s, 3H, 19-C*H_3_*), 2.17 (s, 3H, OCOC*H_3_*), 4.47 (s, 1H, 11α-C*H*), 4.87 (*pseudo*-q, *J* = 17.37, 2H, 21-C*H_2_*OCOCH_3_), 6.00 (s, 4-C*H*), 6.26 (dd, *J_1_* = 1.83 Hz, *J_2_* = 10.09 Hz, 1H, 2-C*H*), 7.26 (d, *J* = 10.08 Hz, 1H, 1-C*H*). ^13^C-NMR (100.6 MHz, CDCl_3_): δ 14.7, 17.0, 20.6, 21.0, 31.2, 32.0, 32.6, 34.0, 35.9, 40.0, 44.2, 48.9, 50.7, 55.5, 68.3, 70.1, 91.2, 122.3, 127.7, 156.8, 170.7, 171.0, 186.8, 205.2.

#### 3.2.4. Synthesis of 17α-Hydroxy-16α-methyl-3,11,20-trioxopregna-1,4-dien-21-acetate (**12**)

To a solution of 11β,17α-dihydroxy-16α-methyl-3,20-dioxopregna-1,4-dien-21-acetate (**11**, 27.4 g, 65.8 mmol) in CH_2_Cl_2_ (270 mL), pyridinium chlorochromate (30.4 g, 141.2 mmol) was added portionwise over 30 min at 0 °C. At the end of the addition, the resulting suspension was stirred at 25 °C for further 4 h. The suspension was filtered on a pad of celite, the filtrate was washed with 5% (*w*/*v*) aqueous solution of Na_2_S_2_O_3_ (2 × 200 mL) and the aqueous phase was extracted with CH_2_Cl_2_ (3 × 200 mL). The combined organic extracts were washed with 3 N aqueous HCl (200 mL), H_2_O (200 mL) and brine (200 mL), dried over anhydrous Na_2_SO_4_ and concentrated under reduced pressure. The crude was purified by crystallization from *n*-hexane/EtOAc (4:1, *v*/*v*, 12 mL g^−1^) affording, after drying (35 °C, 16 h, 100 mmHg), the desired 17α-hydroxy-16α-methyl-3,11,20-trioxo-pregna-1,4-dien-21-acetate (**12**, 21.3 g, 51.4 mmol, 71% isolated yield from **6**, ≥98% purity by q^1^H-NMR) as a white solid (m.p.: 186–188 °C). ^1^H-NMR (400 MHz, CDCl_3_): δ 0.75 (s, 3H, 18-C*H_3_*), 0.96 (d, *J* = 7.24 Hz, 3H, 16α-C*H_3_*), 1.42 (s, 3H, 19-C*H_3_*), 2.15 (s, 3H, OCOC*H_3_*), 4.60–5.02 (m, 2H, 21-C*H_2_*OCOCH_3_), 6.06 (s, 4-C*H*), 6.20 (d, *J* = 10.25 Hz, 1H, 2-C*H*), 7.68 (d, *J* = 10.25 Hz, 1H, 1-C*H*). ^13^C-NMR (100.6 MHz, CDCl_3_): δ 14.5, 15.7, 18.8, 20.4, 31.7, 32.2, 33.6, 36.2, 36.8, 42.4, 49.0, 49.8, 52.1, 60.5, 67.6, 90.3, 124.6, 127.5, 155.4, 166.7, 170.5, 186.4, 204.7, 208.5.

#### 3.2.5. Synthesis of 17α,21-Dihydroxy-16α-methyl-3,11,20-trioxopregna-1,4-diene (**13**)

To a solution of 17α-hydroxy-16α-methyl-3,11,20-trioxo-pregna-1,4-dien-21-acetate (**12**, 21.3 g, 51.4 mmol) in MeOH (500 mL), an aqueous saturated solution of NaHCO_3_ (125 mL) was added. The resulting mixture was stirred at 80 °C for 4 h. The crude mixture was concentrated under reduced pressure and the residue was extracted with EtOAc (3 × 200 mL). The combined organic extracts were washed with H_2_O (200 mL) and brine (200 mL), dried over anhydrous with Na_2_SO_4_ and concentrated under reduced pressure. The desired 17α,21-dihydroxy-16α-methyl-3,11,20-trioxopregna-1,4-diene (**13**, 18.6 g, 49.9 mmol, 97% recovery, ≥90% purity by q^1^H-NMR) was obtained as a white solid and used for the next step without further purification. ^1^H-NMR (400 MHz, CDCl_3_): δ 0.73 (s, 3H, 18-C*H_3_*), 0.97 (d, *J* = 7.24 Hz, 3H, 16α-C*H_3_*), 1.41 (s, 3H, 19-C*H_3_*), 4.19–4.60 (m, 2H, 21-C*H_2_*OH), 6.06 (s, 4-C*H*), 6.20 (dd, *J_1_* = 1.81, *J_2_* = 7.64 Hz, 1H, 2-C*H*), 7.65 (d, *J* = 7.64 Hz, 1H, 1-C*H*). ^13^C-NMR (100.6 MHz, CDCl_3_): δ 14.7, 16.0, 18.7, 31.8, 32.2, 33.5, 36.2, 37.2, 42.4, 49.0, 49.9, 52.5, 60.4, 67.8, 89.6, 124.5, 127.5, 155.4, 166.7, 186.4, 208.6, 211.7.

#### 3.2.6. Synthesis of 21-Chloro-17α-hydroxy-16α-methyl-3,11,20-trioxo-pregna-1,4-diene (**14**)

To a solution of 17α,21-dihydroxy-16α-methyl-3,11,20-trioxo-pregna-1,4-diene (**13**, 18.5 g, 49.7 mmol) in CH_2_Cl_2_ (150 mL), 4-dimethylaminopyridine (12.1 g, 99.3 mmol) was added at 25 °C. The solution was cooled to 0 °C and methanesulfonyl chloride (6.9 mL, 89.1 mmol) was added dropwise over 10 min. After 30 min, the mixture was allowed to warm to 25 °C and stirred for a further 30 min. The mixture was then refluxed and stirred for a further 3 h. The reaction mixture was allowed to cool to room temperature and washed with aqueous saturated solution of NaHCO_3_ (200 mL), 3N aqueous HCl (200 mL), H_2_O (200 mL) and brine (200 mL), dried over anhydrous with Na_2_SO_4_ and concentrated under reduced pressure. The crude 21-chloro-17α-hydroxy-16α-methyl-3,11,20-trioxo-pregna-1,4-diene (**14**, 17.7 g, 45.4 mmol, 92% recovery, ≥85% purity by q^1^H-NMR) was obtained as a white solid and used for the next step without further purification. ^1^H-NMR (400 MHz, CDCl_3_): δ 0.74 (s, 3H, 18-C*H_3_*), 0.97 (d, *J* = 7.24 Hz, 3H, 16α-C*H_3_*), 1.42 (s, 3H, 19-C*H_3_*), 4.16–4.55 (m, 2H, 21-C*H_2_*Cl), 6.07 (s, 4-C*H*), 6.20 (dd, *J_1_* = 1.71, *J_2_* = 10.24 Hz, 1H, 2-C*H*), 7.67 (d, *J* = 7.64 Hz, 1H, 1-C*H*). ^13^C-NMR (100.6 MHz, CDCl_3_): δ 14.6, 16.1, 18.8, 31.7, 32.2, 33.5, 36.2, 37.0, 42.4, 47.8, 48.9, 50.1, 52.2, 60.4, 90.8, 124.6, 127.6, 155.3, 166.6, 186.4, 202.7, 208.4.

#### 3.2.7. Synthesis of 2-Chloro-1-[17′α-((furan-2″-carbonyl)oxy)-16′α-methyl-3′,11′,20′-trioxo-pregna-1′,4′-dienyl)vinyl Furan-2-carboxylate (**15**)

To a solution of 21-chloro-17α-hydroxy-16α-methyl-3,11,20-trioxo-pregna-1,4-diene (**14**, 17.7 g, 45.4 mmol) in CH_2_Cl_2_ (170 mL), Et_3_N (25 mL, 181.1 mmol), dimethylaminopyridine (1.38 g, 11.3 mmol) and 2-furoyl chloride (8.9 mL, 90.6 mmol) were sequentially added at 0 °C and the resulting solution was stirred at 0 °C for 1 h and for a further 2 h at 25 °C. The mixture was washed with 3 N aqueous HCl (200 mL), aqueous saturated solution of NaHCO_3_ (200 mL), H_2_O (200 mL) and brine (200 mL), dried over anhydrous Na_2_SO_4_ and concentrated under reduced pressure. The crude was filtered on a short pad of silica (eluent: *n*-hexane/acetone 7:3, *v*/*v*) affording the desired 2-chloro-1-[17′α-((furan-2″-carbonyl)oxy)-16′α-methyl-3′,11′,20′-tri oxo-pregna-1′,4′-dienyl)vinyl furan-2-carboxylate (**15**, 15.7 g, 27.1 mmol, 57% isolated yield from **12**, ≥96% purity by q^1^H-NMR purity) as a greenish solid (m.p.: 144–146 °C). The product was fully characterized by NOESY and COSY bidimensional NMR experiments, ^1^H- and ^13^C-NMR, and HRMS. ^1^H-NMR (400 MHz, CDCl_3_): δ 1.01 (s, 3H, 18′-C*H_3_*), 1.23 (d, *J* = 7.07 Hz, 3H, 16′α-C*H_3_*), 1.43 (s, 3H, 19′-C*H_3_*), 6.10 (s, 4′-C*H*), 6.19 (d, *J* = 10.26 Hz, 1H, 2′-C*H*), 6.31 (s, 1H, 2-C*H*(Cl)=C), 6.45 (brs, 1H, 4-C*H* furoyl), 6.53 (brs, 1H, 4″-C*H* furoyl), 7.03 (d, *J* = 3.32 Hz, 1H, 3-C*H* furoyl), 7.19 (d, *J* = 3.33 Hz, 1H, 3″-C*H* furoyl), 7.58 (d, *J* = 4.57 Hz, 2H, 5-C*H* + 5″-C*H* furoyl), 7.67 (d, *J* = 10.26 Hz, 1H, 1′-C*H*). ^13^C-NMR (J-mode, 100.6 MHz, CDCl_3_): δ 16.0, 17.4, 18.8, 32.1, 32.6, 33.4, 36.2, 38.4, 42.3, 48.0, 50.3, 53.4, 60.7, 93.7, 111.5, 112.0, 112.1, 118.3, 120.0, 124.8, 127.7, 142.9, 144.65, 144.73, 146.4, 147.7, 152.7, 155.1, 156.4, 166.2, 186.3, 208.1. HRMS *m*/*z* [M + H]^+^ calcd. for C_32_H_31_ClO_8_: 579.1780, found: 579.1785, Δppm: +0.78; *p* + 1 (abund. 31.64%), calcd. 580.1814, found 580.1817, Δppm: +0.57. r.t.: 3.524 min.

#### 3.2.8. Synthesis of 21′-Chloro-(16′α-methyl-3′,11′,20′-trioxo-pregna-1′,4′-dien-17′-yl) Furan-2-carboxylate (mometasone furoate EP impurity C, **2**)

To a solution of 2-chloro-1-[17′α-((furan-2″-carbonyl)oxy)-16′α-methyl-3′,11′,20′-trioxo-pregna-1′,4′-dienyl)vinyl furan-2-carboxylate (**15**, 10.1 g, 17.4 mmol) in CH_2_Cl_2_ (500 mL), HClO_4_ (11 mL) was slowly added dropwise over 30 min at −10 °C. The mixture was stirred at −10 °C for a further 30 min. Aqueous saturated solution of NaHCO_3_ (200 mL) was then slowly added and the phases were separated. The aqueous phase was extracted with CH_2_Cl_2_ (2 × 200 mL), and the combined organic extracts were washed with H_2_O (200 mL) and brine (200 mL), dried over anhydrous with Na_2_SO_4_ and concentrated under reduced pressure. The crude was purified by automated flash chromatography on silica gel (eluent: *n*-hexane/acetone from 100:0 to 70:30, *v*/*v*) and the collected fractions were dried under vacuo and crystallized from acetone/MeOH (1:1, *v*/*v*, 3.7 mL g^−1^) affording, after drying (35 °C, 16 h, 100 mmHg), the desired 21′-chloro-(16′α-methyl-3′,11′,20′-trioxo-pregna-1′,4′-dien-17′-yl) furan-2-carboxylate (mometasone furoate EP impurity C, **2**, 6.2 g, 12.7 mmol, 73% isolated yield) as a white solid. (m.p.: 211 °C, dec.). The product was fully characterized by NOESY and COSY bidimensional NMR experiments, ^1^H- and ^13^C-NMR, IR and HRMS. ^1^H-NMR (600 MHz, CDCl_3_): δ 0.83 (s, 3H, 18′-C*H_3_*), 1.00 (d, *J* = 7.14 Hz, 3H, 16′α-C*H_3_*), 1.45 (s, 3H, 19′-C*H_3_*), 2.97 (d, *J* = 12.30 Hz, 1H, 12′-C*H_2_*_(a)_), 3.40–3.45 (m, 1H, 16′β-C*H*), 4.03–4.11 (m, 2H, 21-C*H_2_*Cl), 6.11 (s, 1H, 4′-C*H*), 6.23 (dd, *J_1_* = 1.76 Hz, *J_2_* = 10.24 Hz, 1H, 2′-C*H*), 6.57 (dd, *J_1_* = 1.64 Hz, *J_2_* = 3.47 Hz, 1H, 4-C*H* furoyl), 7.29 (d, *J* = 2.50 Hz, 1H, 3-C*H* furoyl), 7.64 (brs, 1H, 5-C*H* furoyl), 7.68 (d, *J* = 10.24 Hz, 1H, 1′-C*H*). ^13^C-NMR (J-mode, 150 MHz, CDCl_3_): δ 16.0, 16.8, 18.8, 32.1, 32.8, 33.4, 36.0, 36.7, 42.2, 44.6, 49.1, 50.2, 51.6, 60.4, 96.8, 112.5, 120.2, 124.3, 127.8, 142.8, 147.5, 154.8, 158.0, 165.9, 186.2, 196.1, 207.5. HRMS *m*/*z* [M + H]^+^ calcd. for C_27_H_29_ClO_6_: 485.1725, found: 485.1728, Δppm: +0.58; *p* + 1 (abund. 27.29%), calcd. 486.1759, found 486.1763, Δppm: +0.65. r.t.: 2.977 min. Purity: HPLC-DAD (method EP for mometasone furoate, not calibrated): 96.18% (254 nm, r.t.: 33.442 min); calibrated ^1^H-NMR (12.07 mg of **2** + 1.96 mg of dimethylsulfone as internal standard): 95.8–96.3%; quantitative NP- and RP-18 TLC: ≥96%.

## 4. Conclusions

Mometasone furoate (MF, **1**) is a potent steroidal anti-inflammatory drug (SAID) that, after more than 30 years since its introduction on the market, still represents one of the most prescribed SAIDs and an essential medicine. During its industrial manufacturing, several API-related, process-related or stability-related impurities have been identified and listed in the corresponding monograph of the European *Pharmacopoeia*. Among them, mometasone furoate EP impurity C (**2**) is commercially available as a reference standard (mg scale) at very high costs, but no synthetic procedure for its preparation has been reported so far. Considering the need to synthesise the target impurity **2** at the gram scale and in high purity grade for regulatory activities, in this work we successfully developed a straightforward multi-gram scale synthesis of mometasone furoate EP impurity C (**2**) starting from cheap and readily available steroidal precursor **6**. The structural elucidation of the target product **2** was discussed along with the synthetic efforts aimed at optimizing both the acylation step and the acid-promoted hydrolysis of difuroate enol ether intermediate **15** to the target impurity **2**. The proposed route resulted in good overall yield, minimized time-consuming and expensive purification extra-steps and a suitable purity of the final product, and it is therefore well suited to be applicable in R&D laboratories of steroid pharmaceutical companies.

## Figures and Tables

**Table 1:** Screening for the furoylation reaction of intermediate **14**.

Entry	Reagents (Equiv.)	Temperature (°C)	Time (h)	Isolated Yield (%)
1 *^a^*	Pyridine (2), 2-furoyl chloride (1.2)	25	24	27
2 *^a^*	Et_3_N (2), DMAP (0.1), 2-furoyl chloride (1.2)	25	24	46
3 *^b^*	Et_3_N (2), DMAP (0.1), 2-furoyl chloride (1.2)	reflux	48	48
4 *^b^*	Et_3_N (4), DMAP (0.25), 2-furoyl chloride (2)	25	3.5	76 *^c^*

*^a^* Reaction performed at 100 mg scale. *^b^* Reaction performed at 1 g scale. *^c^* Yield refers to difuroate enol ether **15**.

**Table 2:** Screening for the conversion of difuroate enol ether **15** to target **2**.

Entry *^a^*	Reagents (Amount)	Temperature (°C)	Solvent and (15)	Results
1	12 N HCl (1 mL/mmol)	25	1,4-dioxane (0.25 M)	Side products formation
2	12 N HCl (1 mL/mmol)	25	AcOH (0.25 M)	Side products formation
3	12 N HCl (0.1 mL/mmol)	25	AcOH (0.25 M)	Side products formation
4	3 N HCl (2 mL/mmol)	0	AcOH (0.25 M)	Traces of **2**
5	*p*TSA (2 equiv.)	25, then reflux	H_2_O/Acetone (0.25 M)	Traces of **2**
6	HClO_4_ (0.2 mL/mmol)	0	CH_2_Cl_2_ (0.25 M)	46% yield
7	HClO_4_ (1 mL/mmol)	−10	CH_2_Cl_2_ (0.025 M)	72% yield

*^a^* Reactions were performed at 100–150 mg scale for 30–60 min. [15]: Molar concentration of substrate **15** in the solvent system.

**Table 3:** Comparison of the results obtained for the preparation of mometasone furoate EP impurity C (**2**) at mg and multi-gram scale.

Step	Mg Scale Synthesis *^a^*			Multi-Gram Scale Synthesis *^b^*		
	Work-Up Procedure	Purification	Yield (Purity) *^c^*	Work-Up Procedure	Purification	Yield (Purity) *^c^*
*a*	Extraction	None	95% *^d^* (≥95%)	Precipitation	None	99% *^d^* (≥97%)
*b*	Extraction	None	90% *^d^* (≥90%)	Precipitation	None	93% *^d^* (≥90%)
*c*	Extraction	Chromatography	82% *^e^* (≥95%)	Precipitation	None	99% *^d^* (≥87%)
*d*	Extraction	Chromatography	74% *^e^* (≥95%)	Extraction	Crystallization	71% *^e^* (≥98%)
*e*	Extraction	None	95% *^c^* (≥90%)	Extraction	None	97% *^c^* (≥90%)
*f*	Extraction	None	92% *^c^* (≥90%)	Extraction	None	92% *^c^* (≥85%)
*g*	Extraction	Chromatography	76% *^e^* (≥90%)	Extraction	Silica filtration	57% *^e^* (≥96%)
*h*	Extraction	Chromatography	72% *^e^* (≥90%)	Extraction	Chromatography + Crystallization	73% *^e^* (≥96%) *^f^*
**Overall yield**	**33%**			**30%**		

*^a^* Starting from 500 mg of **6**. *^b^* Starting from 27 g of **6**. *^c^* Esteemed by ^1^H-NMR analysis. *^d^* Crude recovery. *^e^* Isolated yield. *^f^* Determined by quantitative NP- and RP_18_-TLC, HPLC-DAD analysis and quantitative ^1^H-NMR in the presence of dimethyl sulfone (standard for quantitative NMR, TraceCERT^®^) as the standard (see Supplementary Data).

## Data Availability

Data are contained within the article and Supplementary Materials.

## Supplementary Materials

The following supporting information can be downloaded at: https://www.mdpi.com/article/10.3390/molecules28237859/s1, analytical and spectroscopic characterization of compounds **2**, **9**–**15**.
