# Supplementary material for: Multi-Gram Scale Synthesis and Characterization of Mometasone Furoate EP Impurity C
Source: Molecules. 2023 Nov 30;28(23):7859. doi: 10.3390/molecules28237859 (PMC10708511; doi:10.3390/molecules28237859)

Multi-Gram Scale Synthesis and Characterization of Mometasone Furoate EP Impurity C

Riccardo Ronchetti [1], Luigi Alfonso Pannone [1], Bruno Cerra [1,*], Emidio Camaioni [1], Gianfranco Lopopolo [2], Emanuele Attolino [2] and Antimo Gioiello [1]

[1] Laboratory of Medicinal and Advanced Synthetic Chemistry (Lab MASC), Department of Pharmaceutical Sciences, University of Perugia, Via del Liceo 1, 06122 Perugia, Italy; riccardo.ronchetti@chimfarm.unipg.it (R.R.); luigialfonso.pannone@studenti.unipg.it (L.A.P.); emidio.camaioni@unipg.it (E.C.); antimo.gioiello@unipg.it (A.G.)

[2] Research & Development Department, Newchem SpA, Via Roveggia, 47, 37136 Verona, Italy; gianfranco.lopopolo@newchemspa.it (G.L.); emanuele.attolino@newchemspa.it (E.A.)

* Correspondence: bruno.cerra@unipg.it

NMR spectra

^{1}H-NMR (400 MHz, $CDCl_3$) of 9β,11β-epoxy-17α-hydroxy-16α-methyl-3,20-dioxo- pregna-1,4-dien-21-acetate (9)

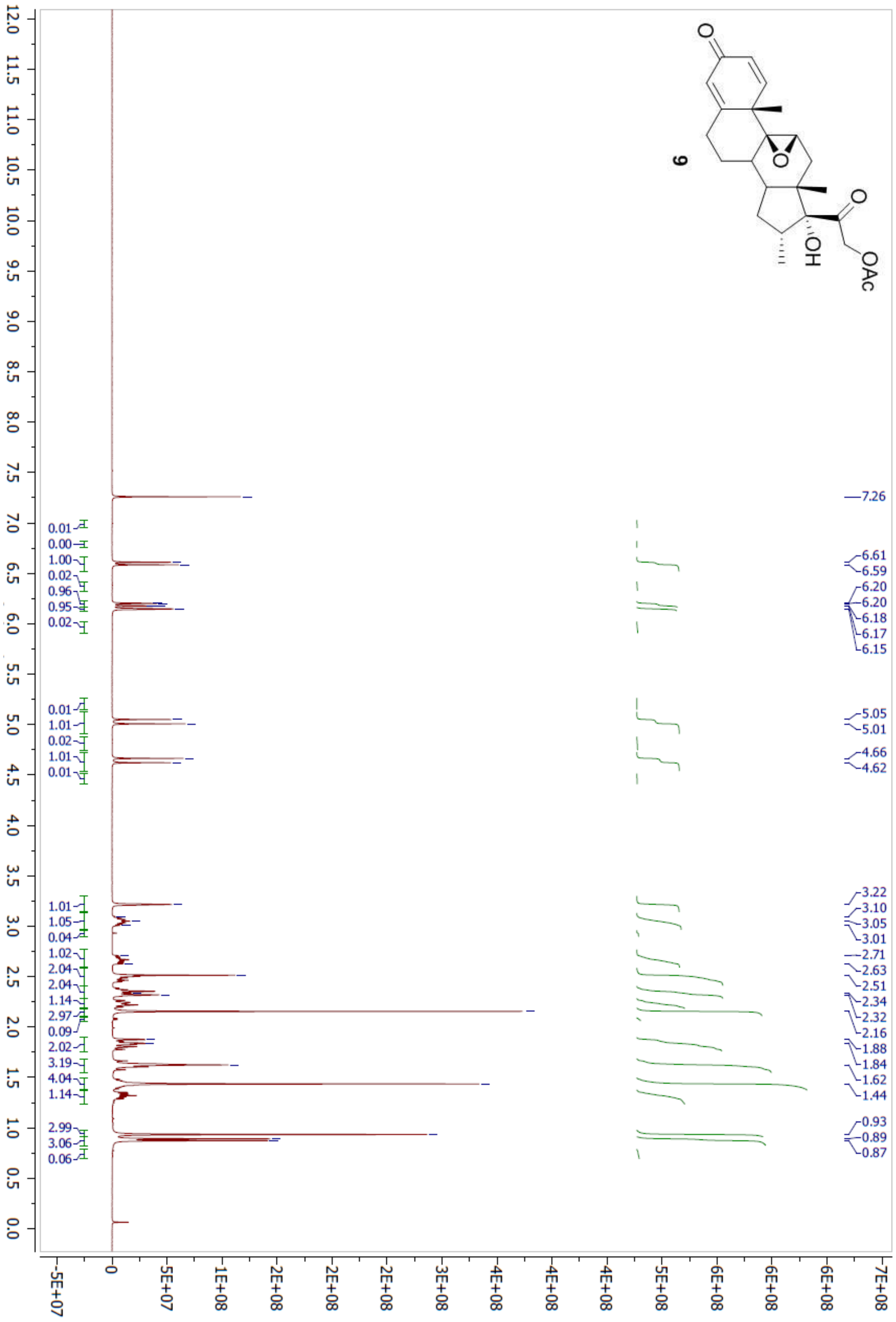

^{13}C-NMR (100.6 MHz, CDCl$_3$) of of 9β,11β-epoxy-17α-hydroxy-16α-methyl-3,20-dioxo- pregna-1,4-dien-21-acetate (9)

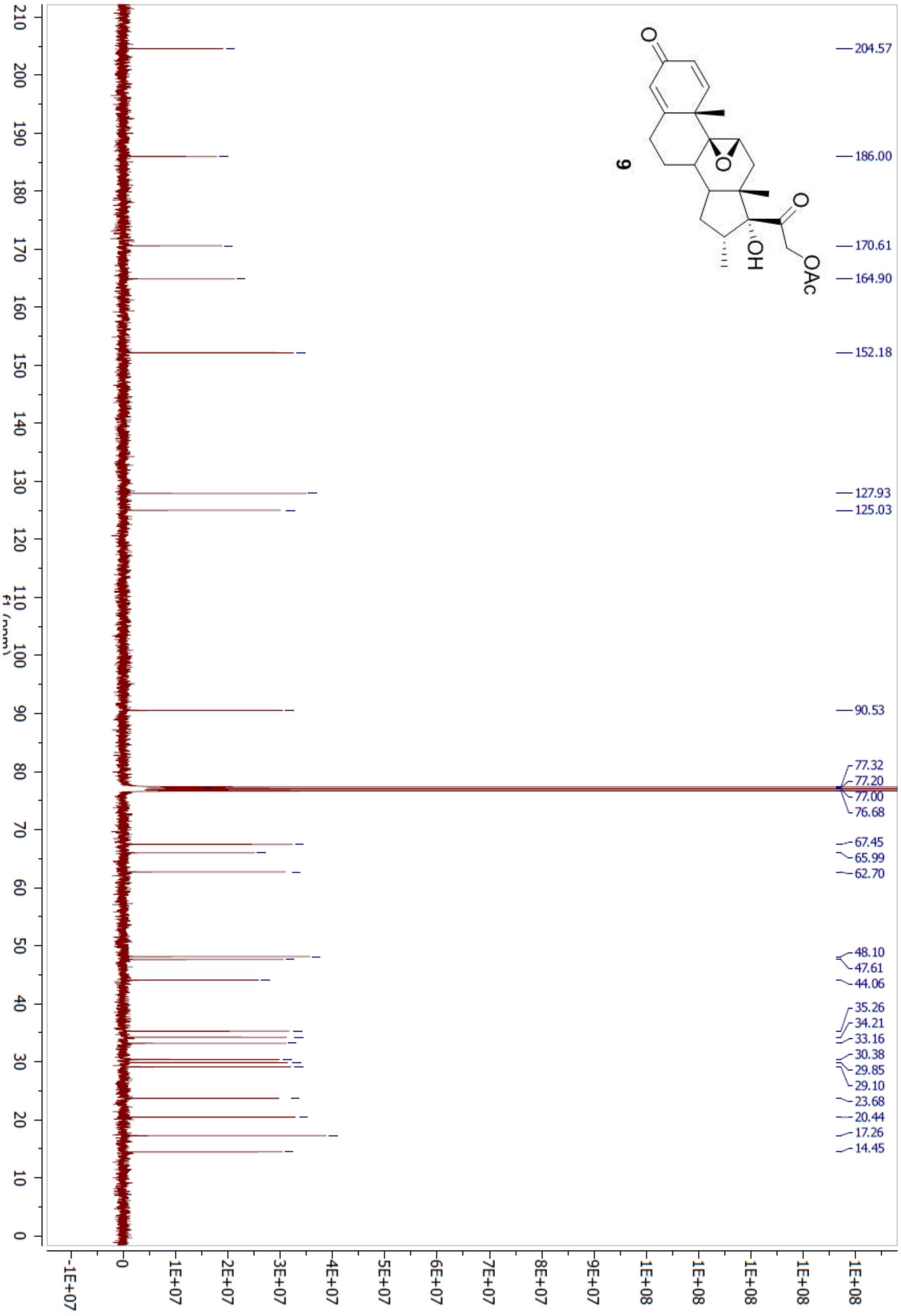

^{1}H-NMR (400 MHz, $CDCl_3$) of 9α-bromo-11β,17α-dihydroxy-16α-methyl-3,20-dioxo-pregna-1,4-dien-21-acetate (10)

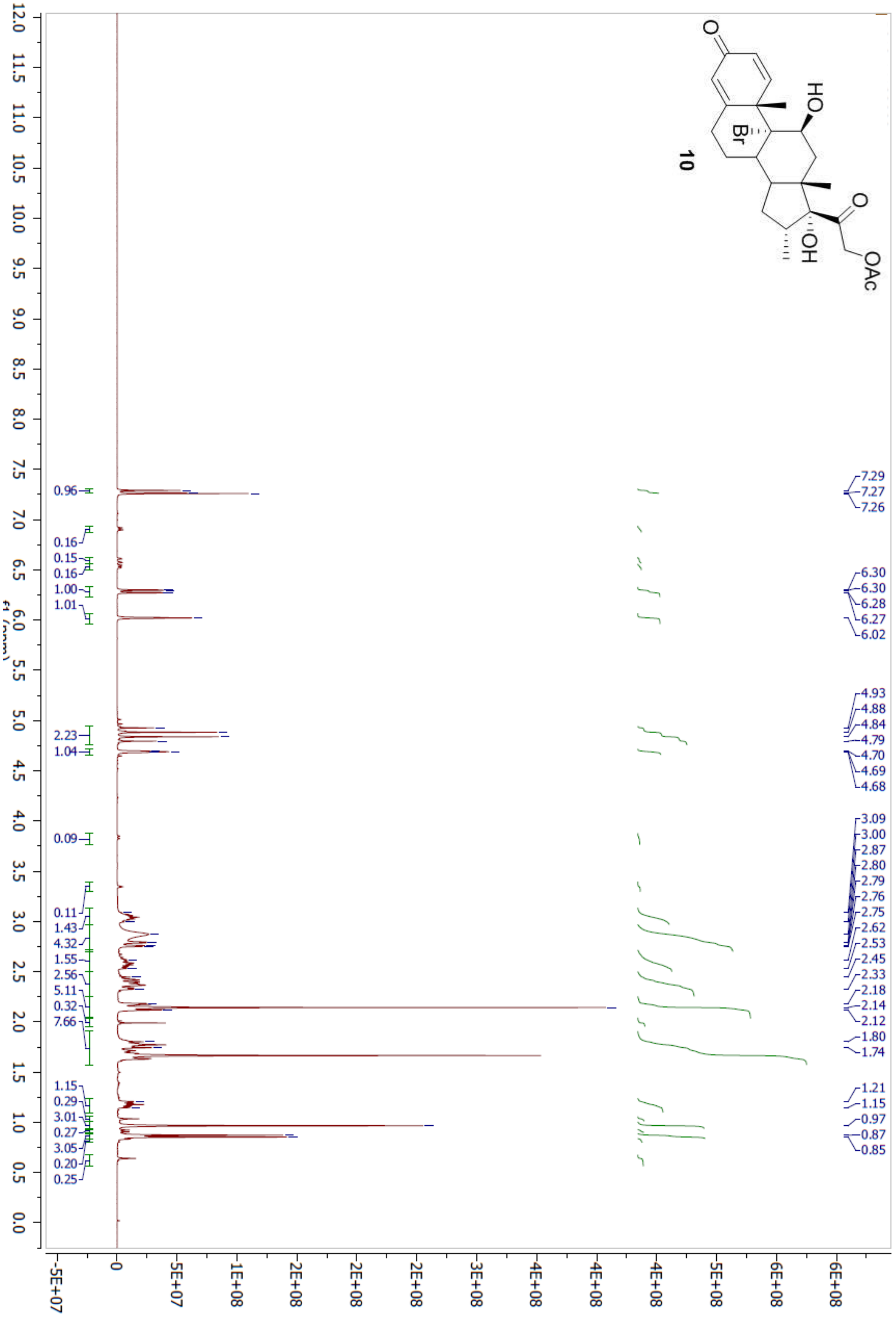

^{13}C-NMR (100.6 MHz, $CDCl_3$) of 9α-bromo-11β,17α-dihydroxy-16α-methyl-3,20-dioxo-pregna-1,4-dien-21-acetate (10)

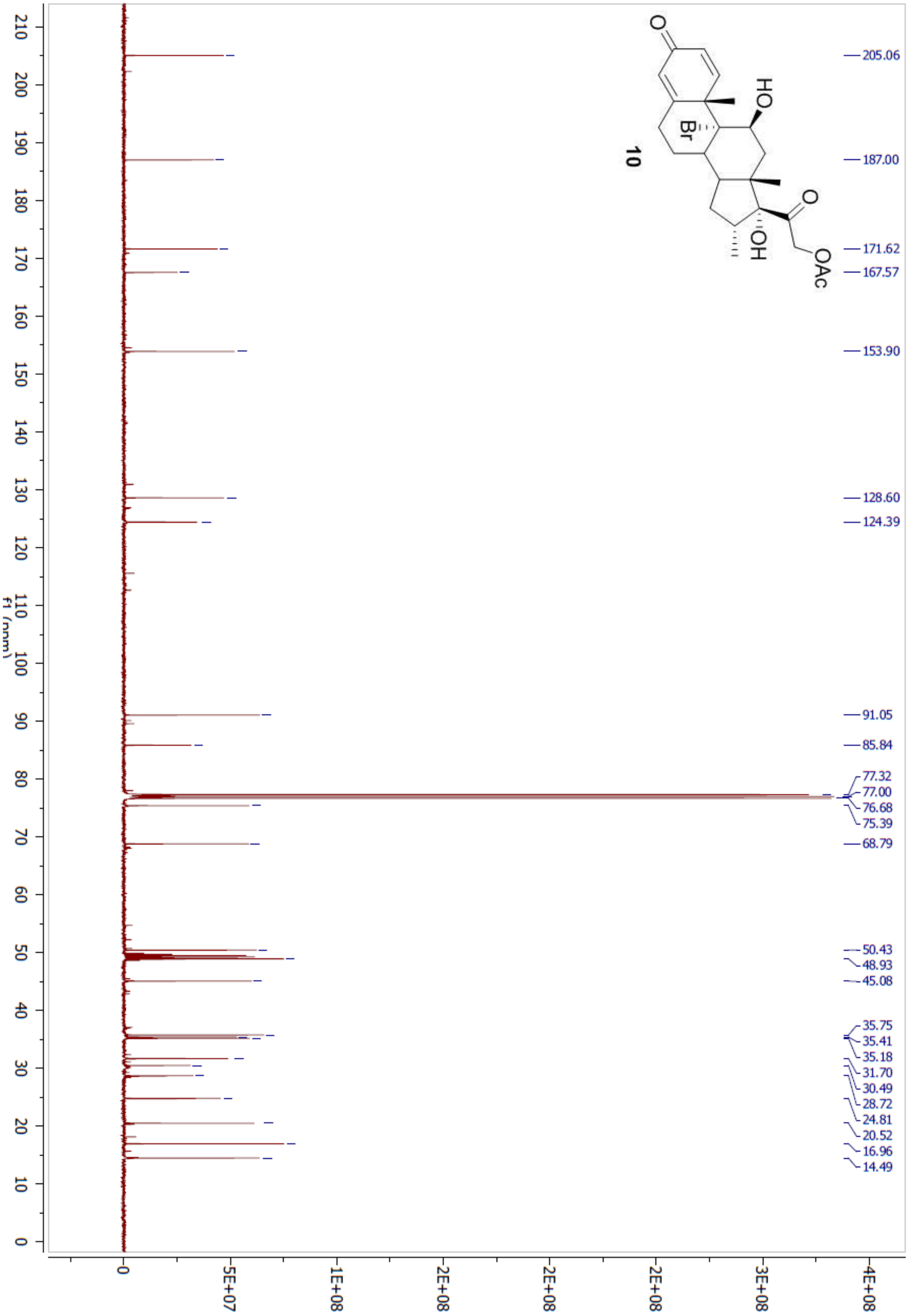

^{1}H-NMR (400 MHz, $CDCl_3$) of 11β,17α-dihydroxy-16α-methyl-3,20-dioxopregna-1,4-dien-21-acetate (11)

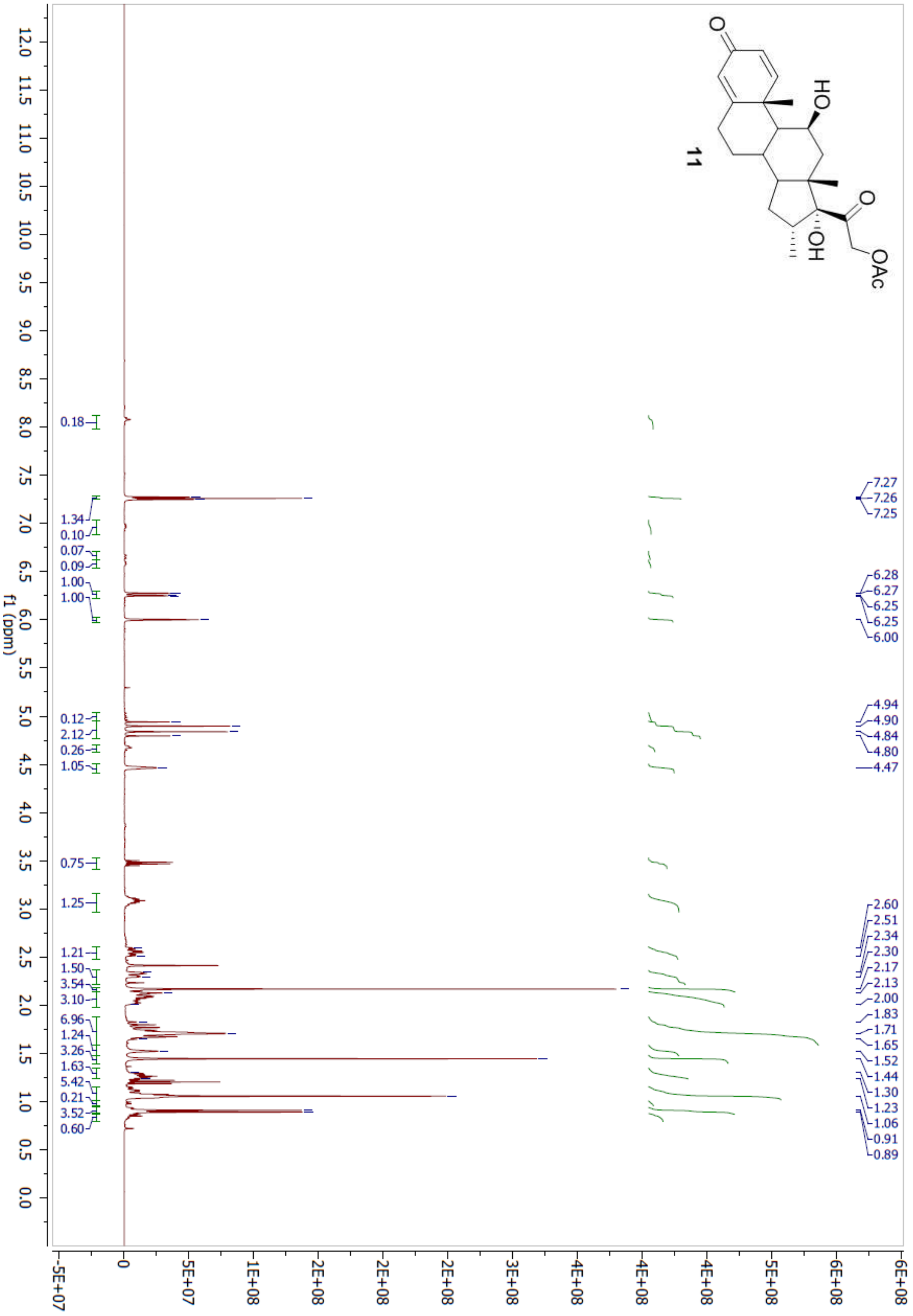

^{13}C-NMR (100.6 MHz, $CDCl_3$) of 11β,17α-dihydroxy-16α-methyl-3,20-dioxopregna-1,4-dien-21-acetate (11)

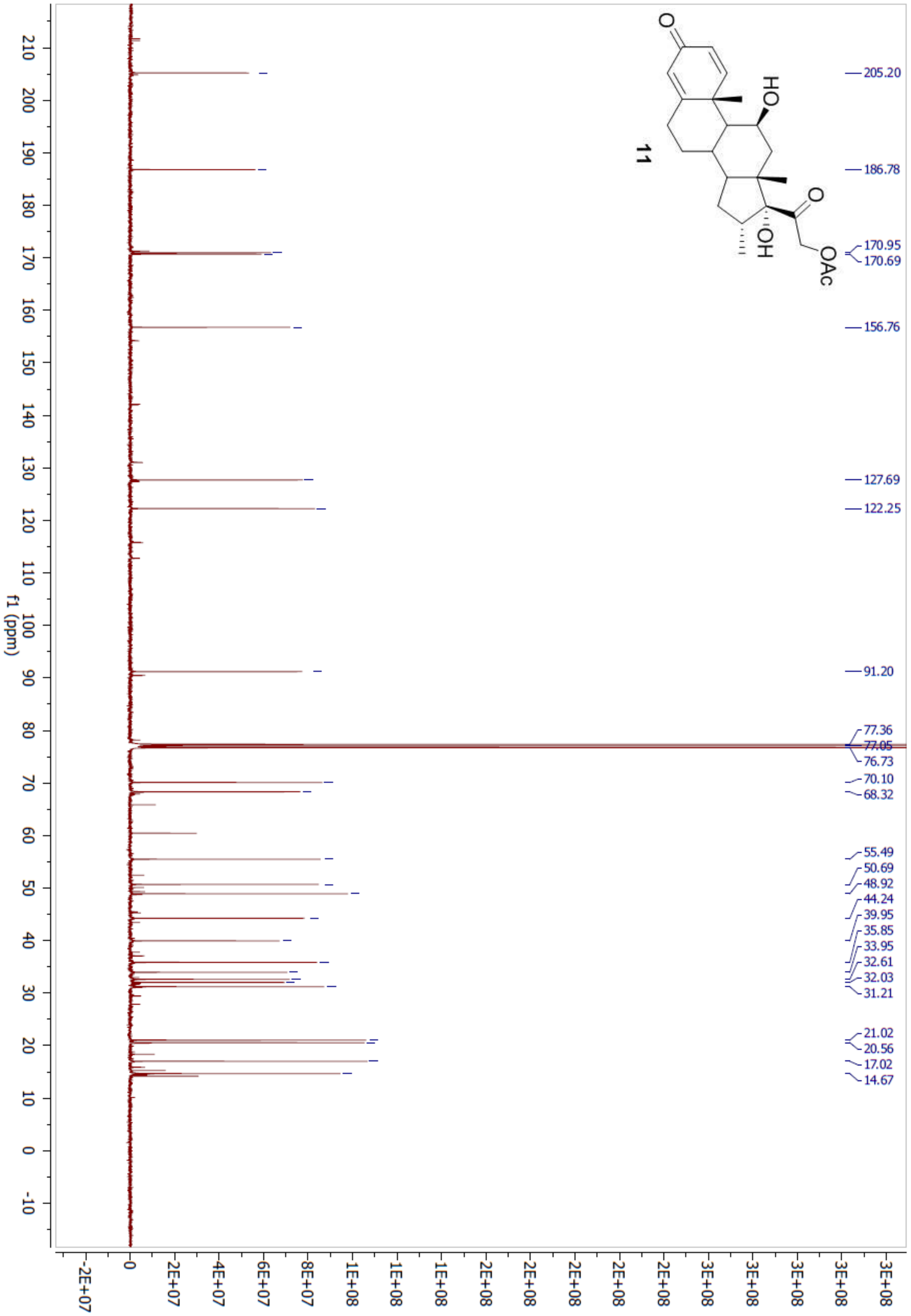

^{1}H-NMR (400 MHz, $CDCl_3$) of 17α-hydroxy-16α-methyl-3,11,20-trioxopregna-1,4-dien-21-acetate (12)

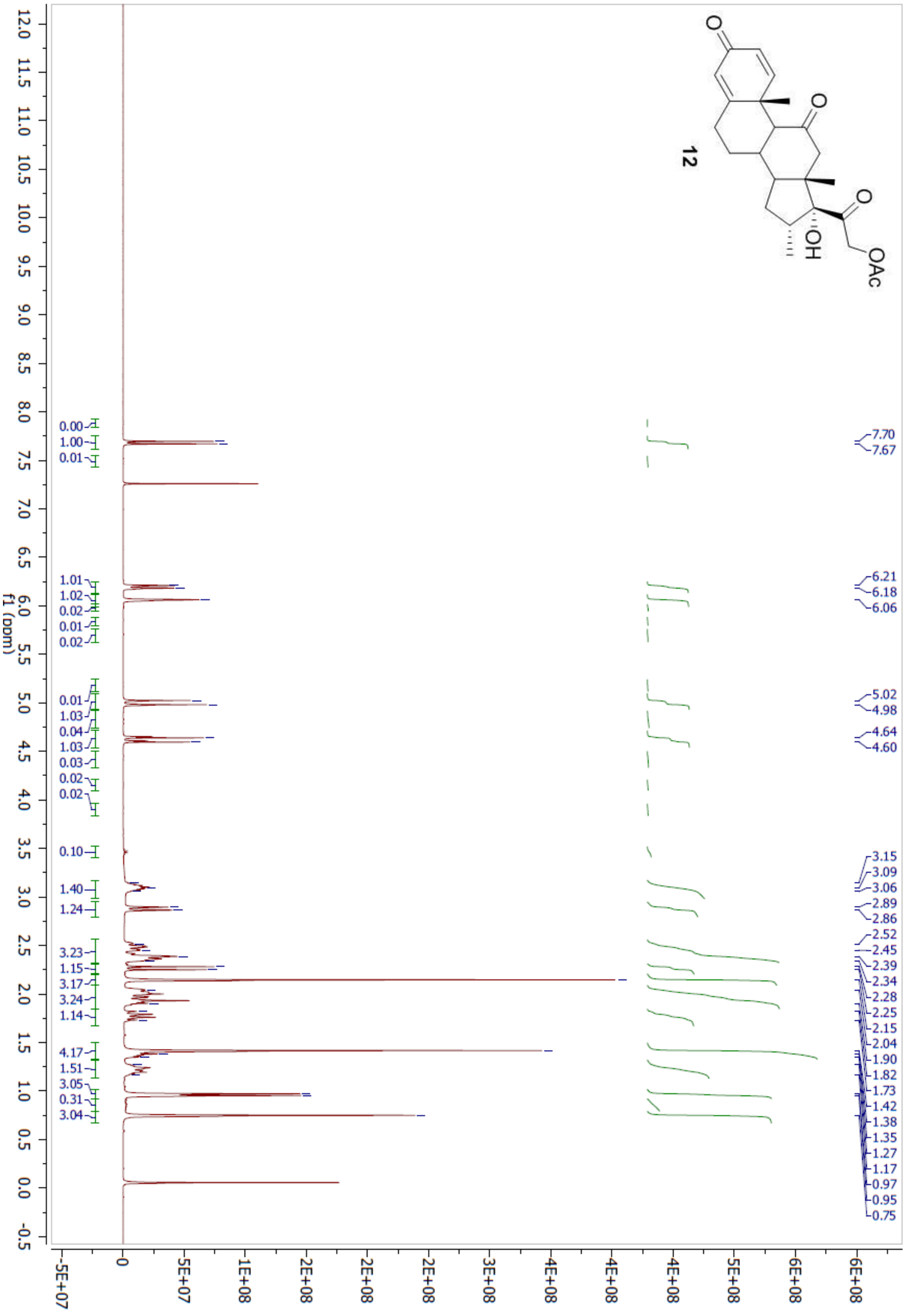

^{13}C-NMR (100.6 MHz, $CDCl_3$) of 17α-hydroxy-16α-methyl-3,11,20-trioxopregna-1,4-dien-21-acetate (12)

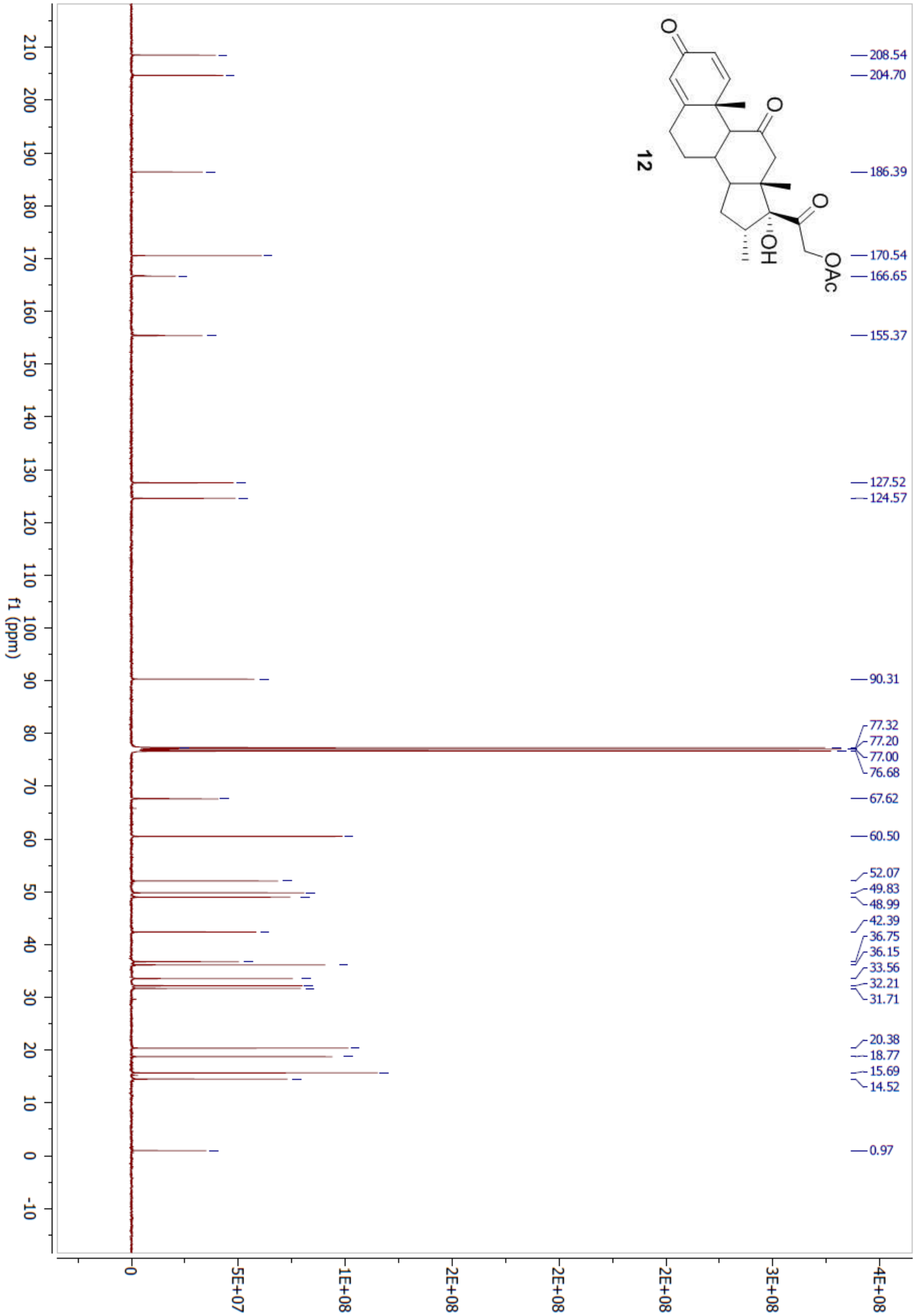

^{1}H-NMR (400 MHz, $CDCl_3$) of 17α,21-dihydroxy-16α-methyl-3,11,20-trioxopregna-1,4-diene (13)

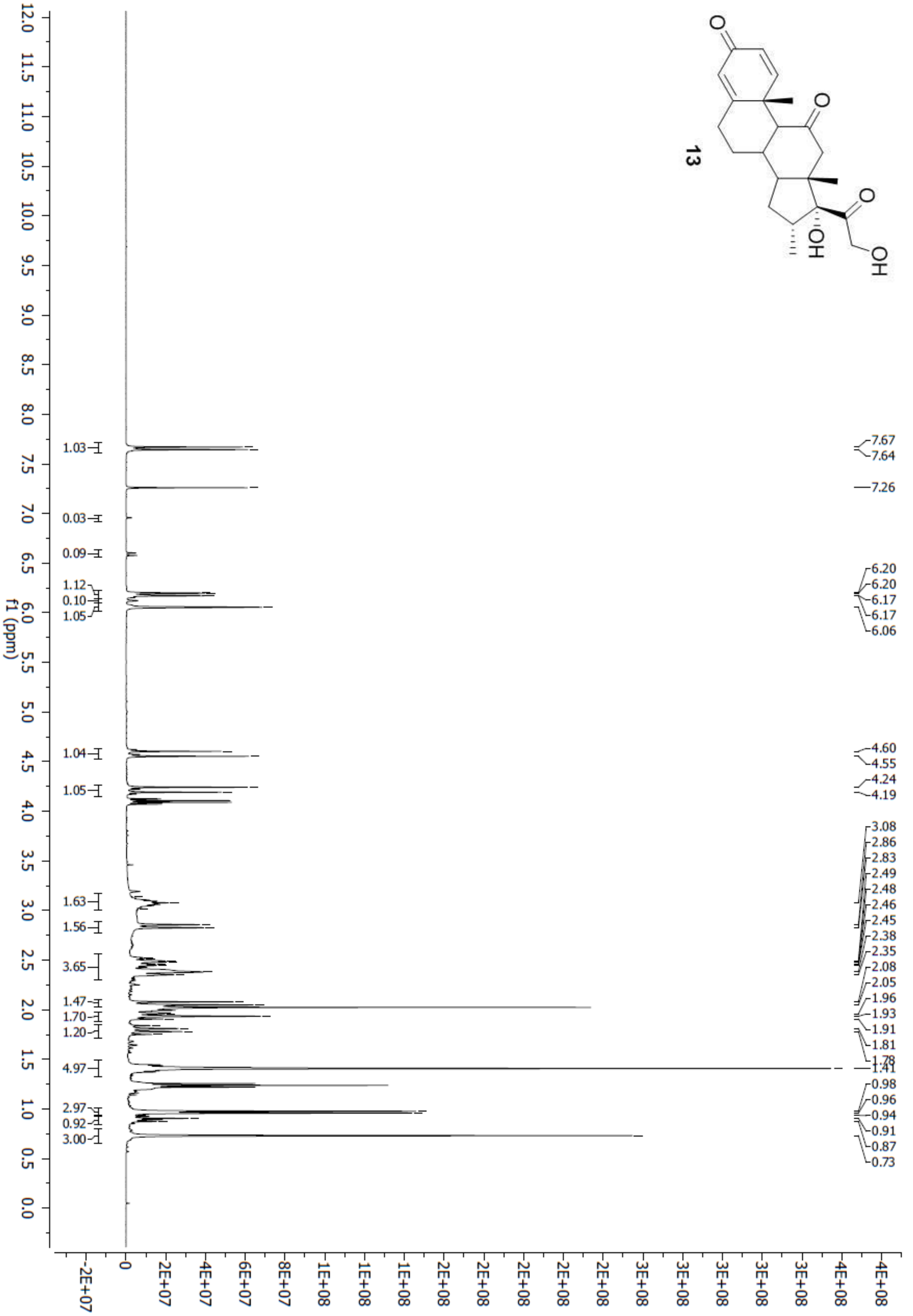

^{13}C-NMR (100.6 MHz, $CDCl_3$) of 17α,21-dihydroxy-16α-methyl-3,11,20-trioxopregna-1,4-diene (13)

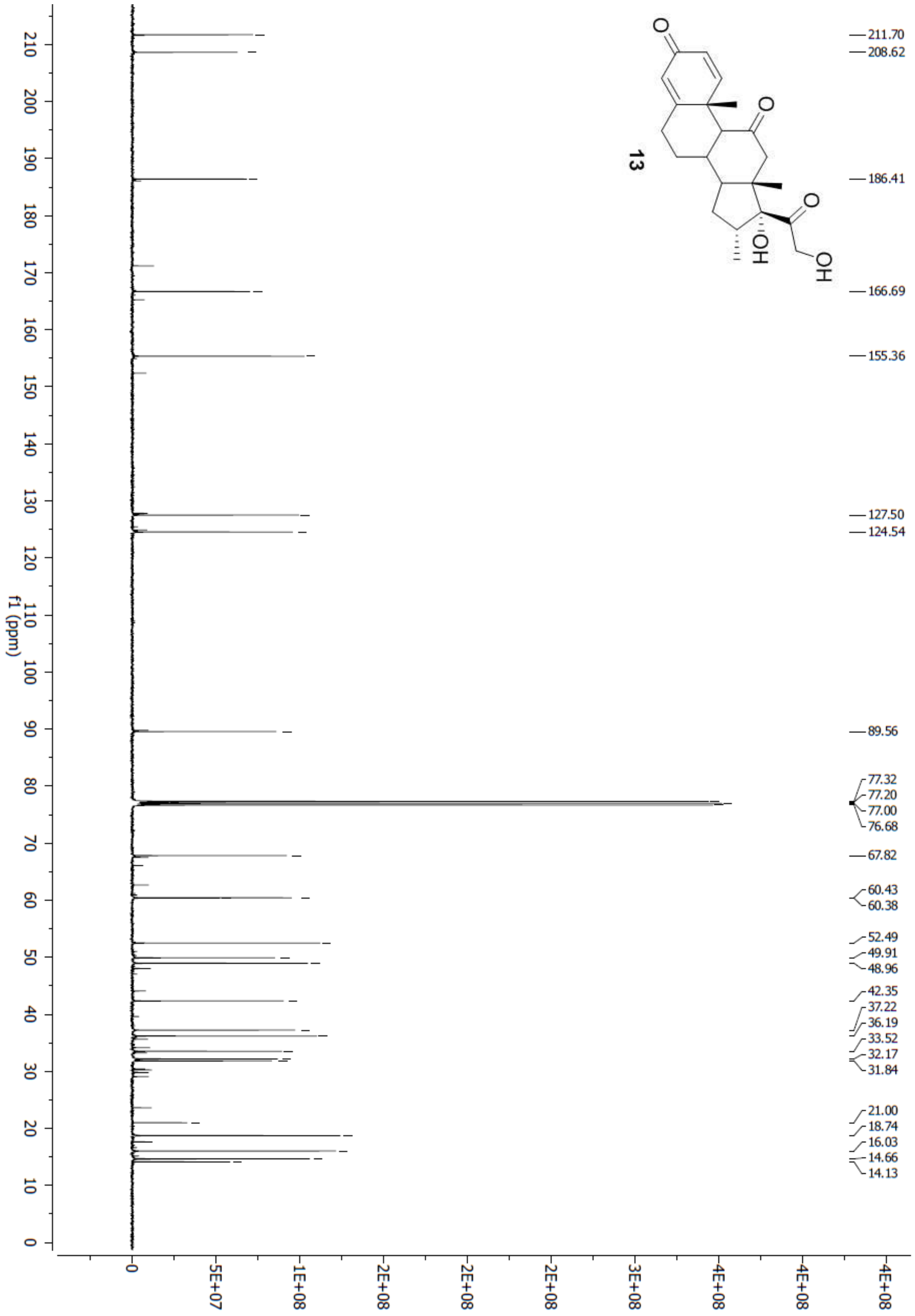

^{1}H-NMR (400 MHz, CDCl$_3$) of 21-chloro-17α-hydroxy-16α-methyl-3,11,20-trioxopregna-1,4-diene (14)

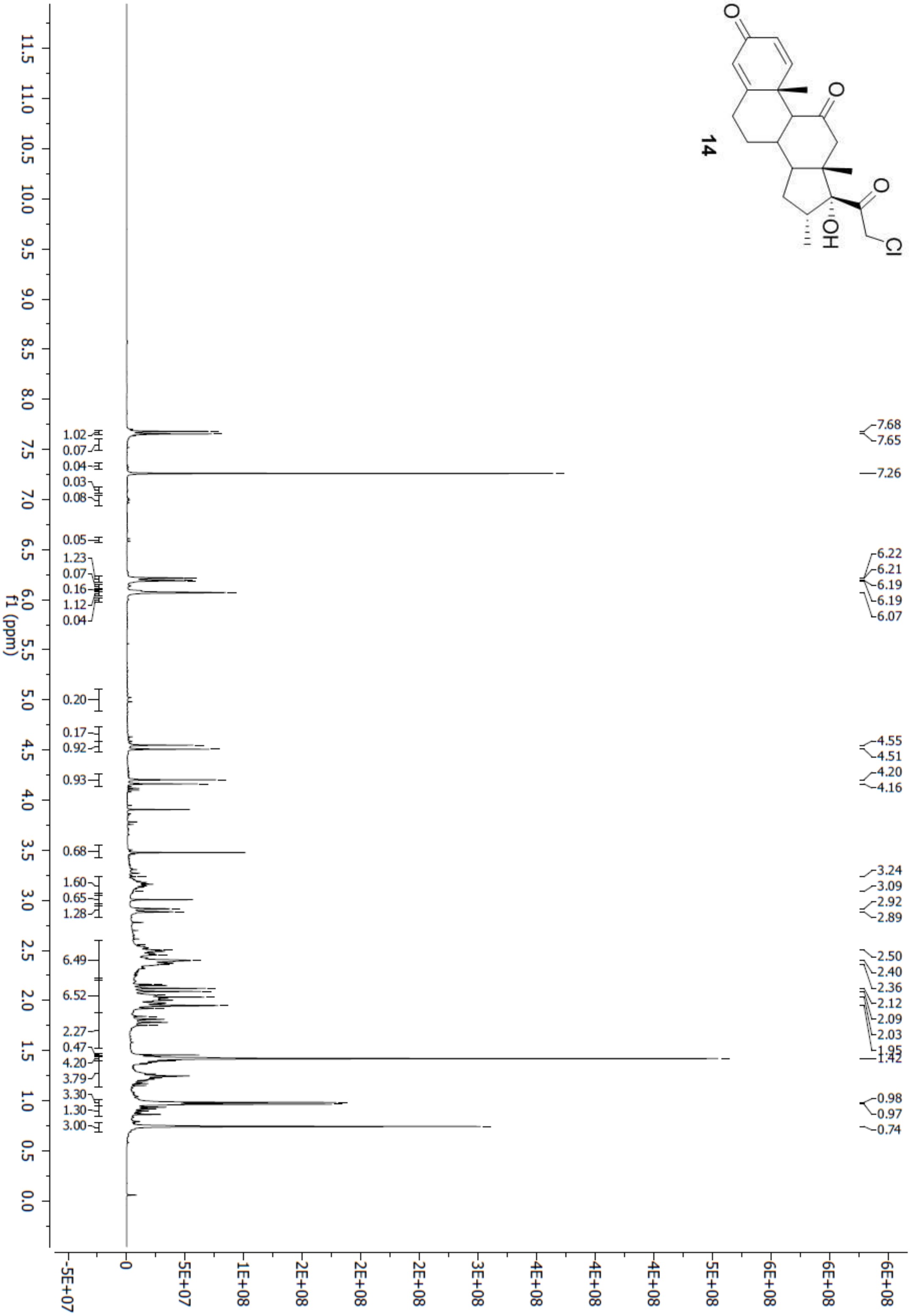

^{13}C-NMR (100.6 MHz, CDCl$_3$) of 21-chloro-17α-hydroxy-16α-methyl-3,11,20-trioxopregna-1,4-diene (14)

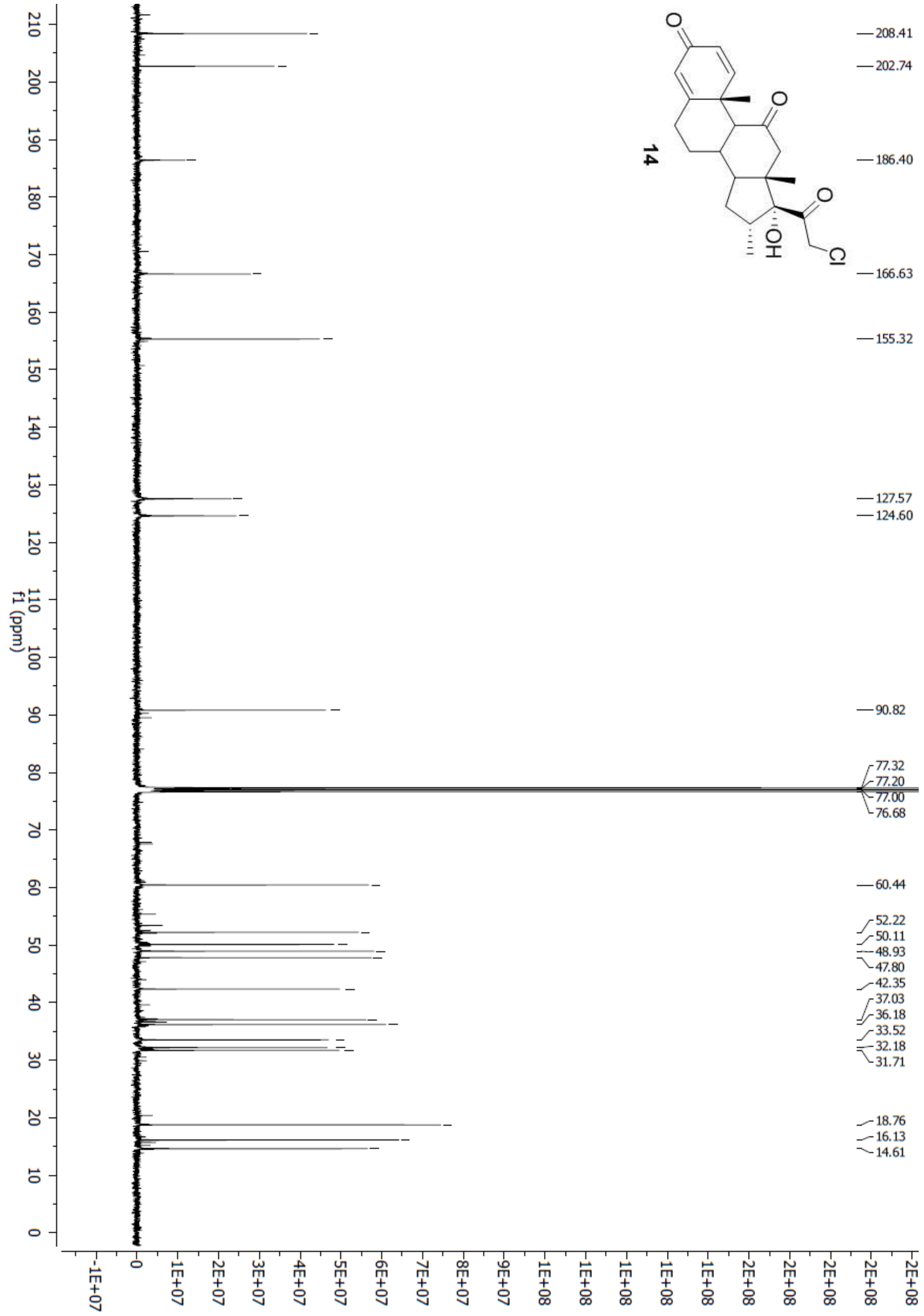

^{1}H-NMR (400 MHz, $CDCl_3$) of 2-chloro-1-[17’α-((furan-2’’-carbonyl)oxy)-16’α-methyl-3’,11’,20’-trioxo-pregna-1’,4’-dienyl)vinyl furan-2-carboxylate (15)

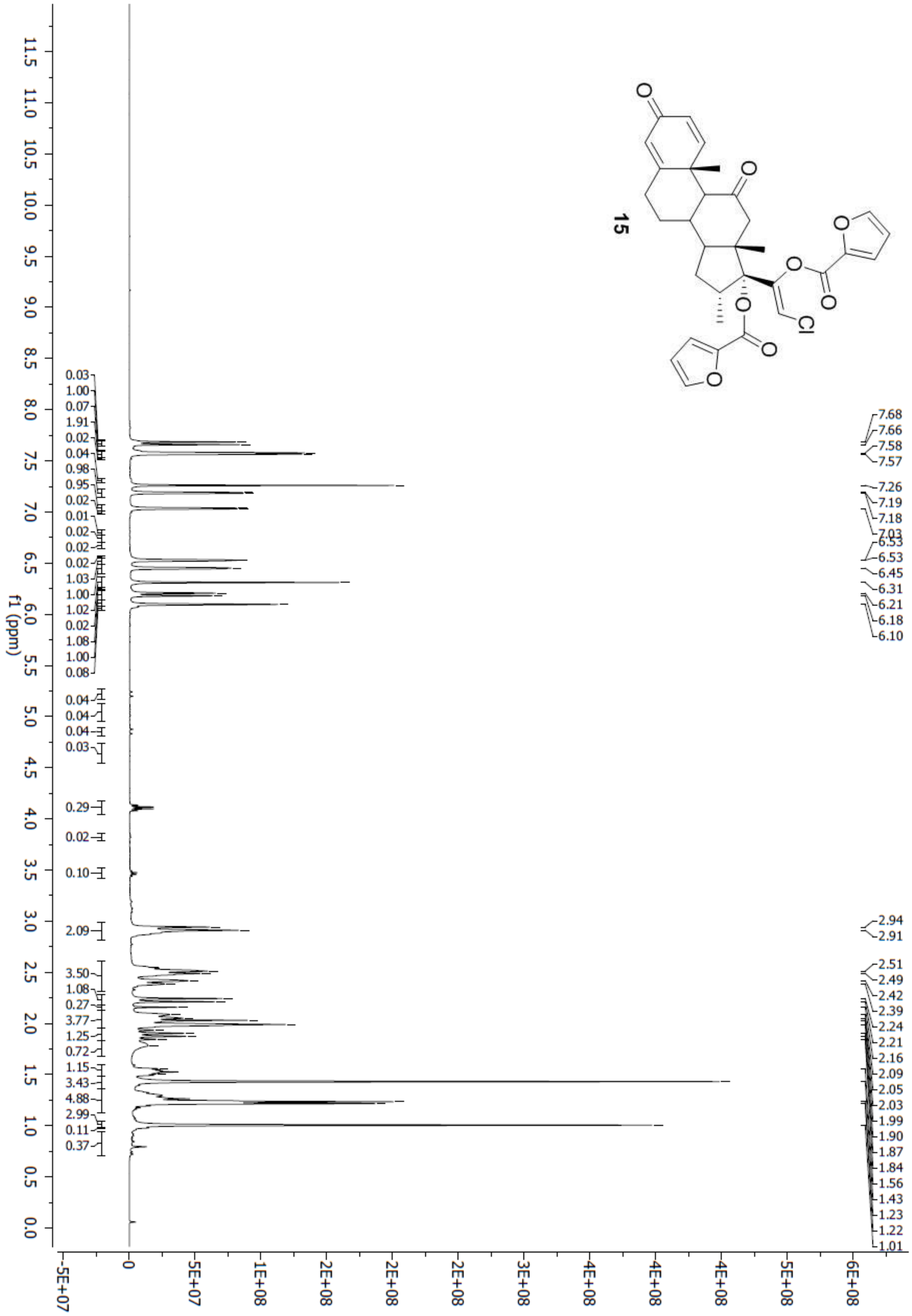

^{13}C-NMR (J-Mode, 100.6 MHz, $CDCl_3$) of 2-chloro-1-[17'α-((furan-2''-carbonyl)oxy)-16'α-methyl-3',11',20'-trioxo-pregna-1',4'-dienyl)vinyl furan-2-carboxylate (15)

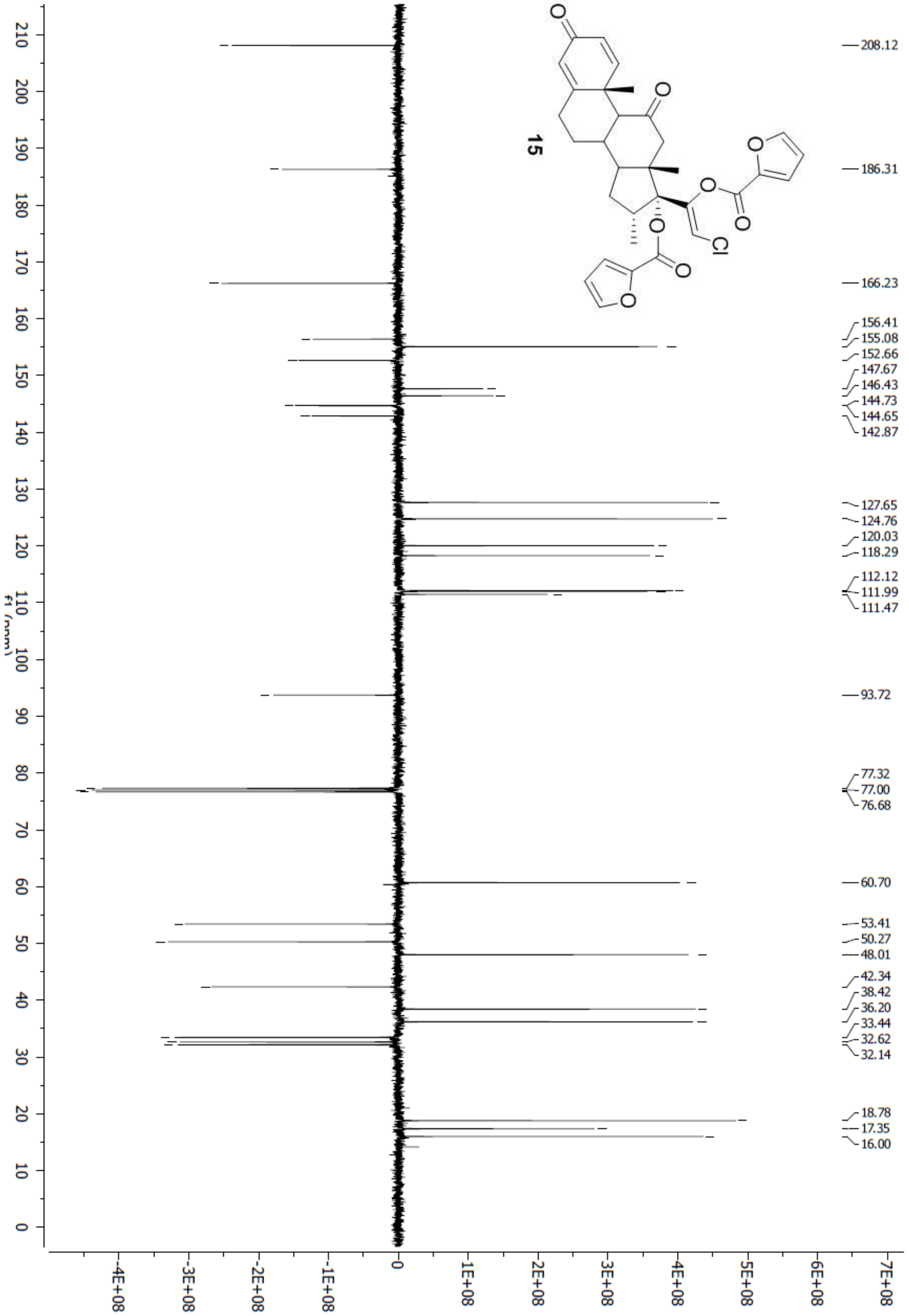

COSY (400 MHz, $CDCl_3$) of 2-chloro-1-[17'α-((furan-2''-carbonyl)oxy)-16'α-methyl-3',11',20'-trioxo-pregna-1',4'-dienyl)vinyl furan-2-carboxylate (15)

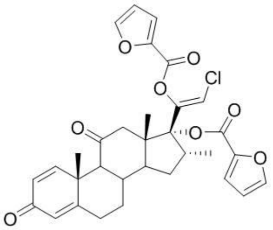

15

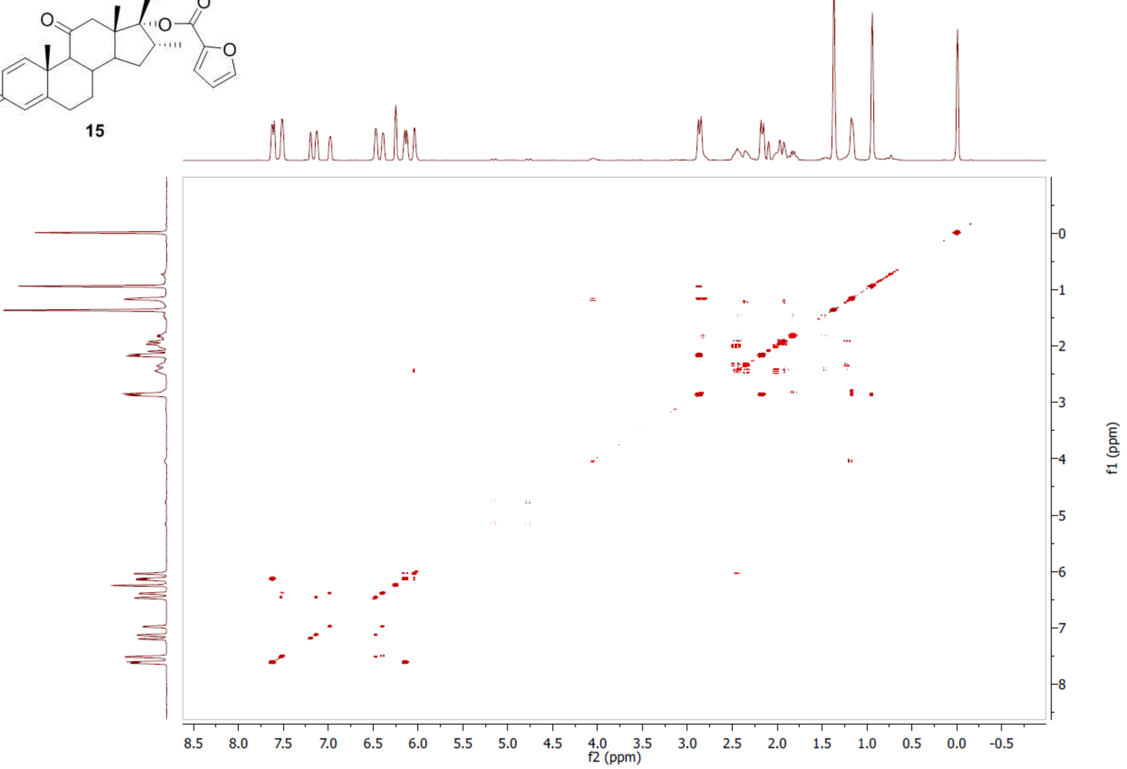

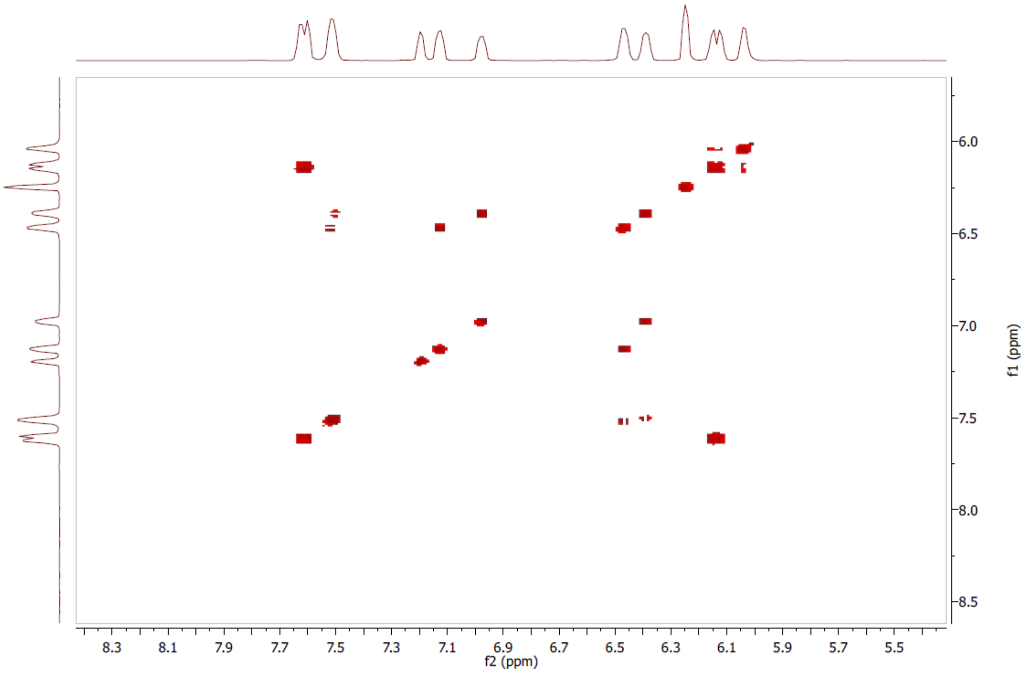

NOESY (400 MHz, $CDCl_3$) of 2-chloro-1-[17'α-((furan-2''-carbonyl)oxy)-16'α-methyl-3',11',20'-trioxo-pregna-1',4'-dienyl)vinyl furan-2-carboxylate (15)

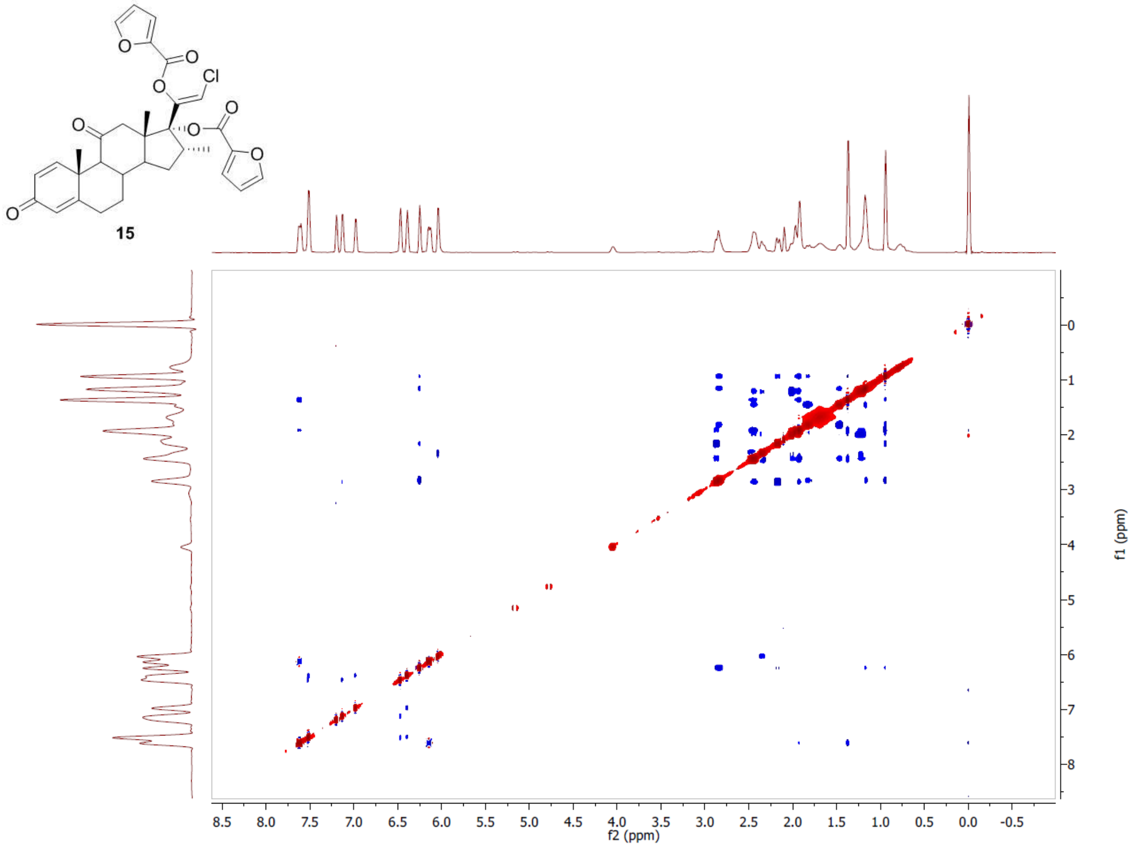

15

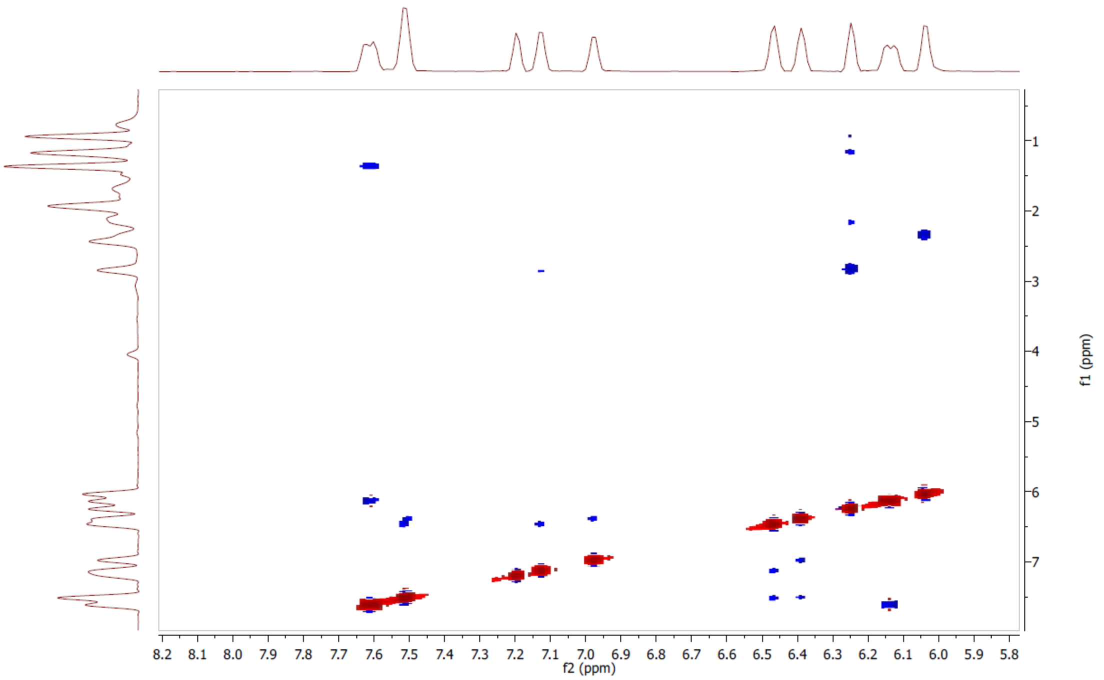

HRMS of 2-chloro-1-[17'α-((furan-2''-carbonyl)oxy)-16'α-methyl-3',11',20'-trioxo-pregna-1',4'-dienyl)vinyl furan-2-carboxylate (15)

Analysis Report

Sample Information

**Name**	MomDiFur BC 31012023	**Data File Path**	D:\MassHunter\Data\Data\Gioiello\MomDiFur BC v3 31012023.d
**Sample ID**		**Acq. Time (Local)**	2/2/2023 1:56:15 PM (UTC+01:00)
**Instrument**	Instrument 1	**Method Path (Acq)**	D:\MassHunter\Methods\ACCURATE MASS DAD 31012023.m
**MS Type**	QTOF	**Version (Acq SW)**	6200 series TOF/6500 series Q-TOF B.09.00 (B9044.1 SP1)
**Inj. Vol. (ul)**	5	**IRM Status**	Success
**Position**	P1-D4	**Method Path (DA)**	D:\MassHunter\Data\Data\Gioiello\MomDiFur BC v3 31012023.d\Results\Qual\Version4\Default_KBr.m
**Plate Pos.**		**Target Source Path**	
**Operator**		**Result Summary**	

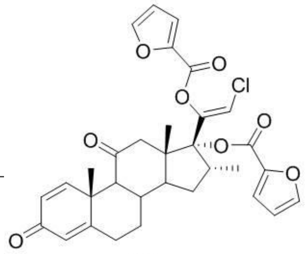

15

Sample Chromatograms

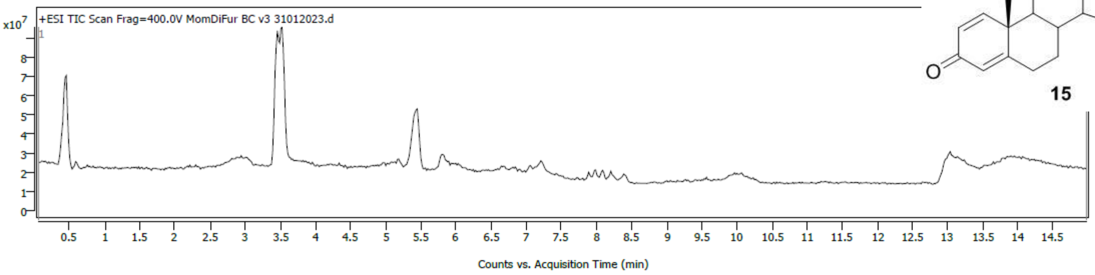

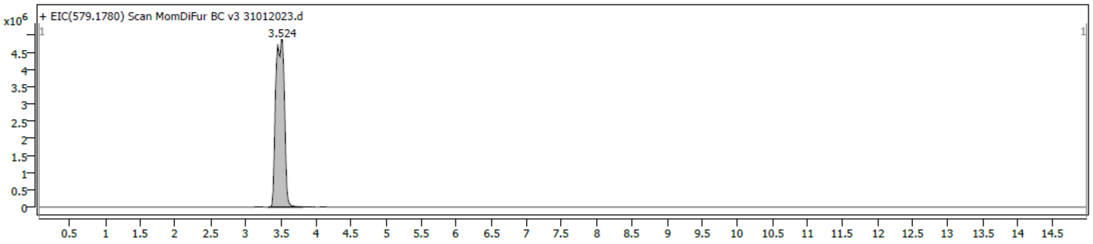

Sample Spectra

+ Scan (rt: 3.474 min) **C32 H31 Cl O8**

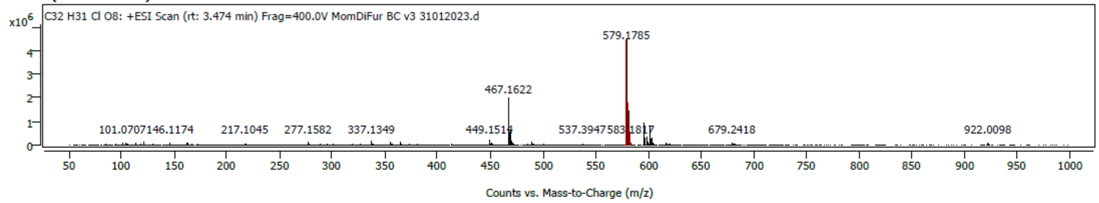

Spectrum Peaks

m/z	Z	Abund	Abund %	m/z (Calc)	Diff (ppm)	Ion Species	Formula	Ion Type
579.1785	1	4464430	100.00	579.1780	0.78	(M+H)+	C32 H31 Cl O8	
580.1817	1	1412346	31.64	580.1814	0.57	(M+H)+	C32 H31 Cl O8	
581.1774	1	1454750	32.59	581.1769	0.91	(M+H)+	C32 H31 Cl O8	
582.1797	1	464053	10.39	582.1793	0.64	(M+H)+	C32 H31 Cl O8	
[illegible]		[illegible]	[illegible]					

^{1}H-NMR (600 MHz, $CDCl_3$) of 21'-chloro-(16'α-methyl-3',11',20'-trioxo-pregna-1',4'-dien-17'-yl) furan-2-carboxylate (mometasone furoate EP impurity C, 2)

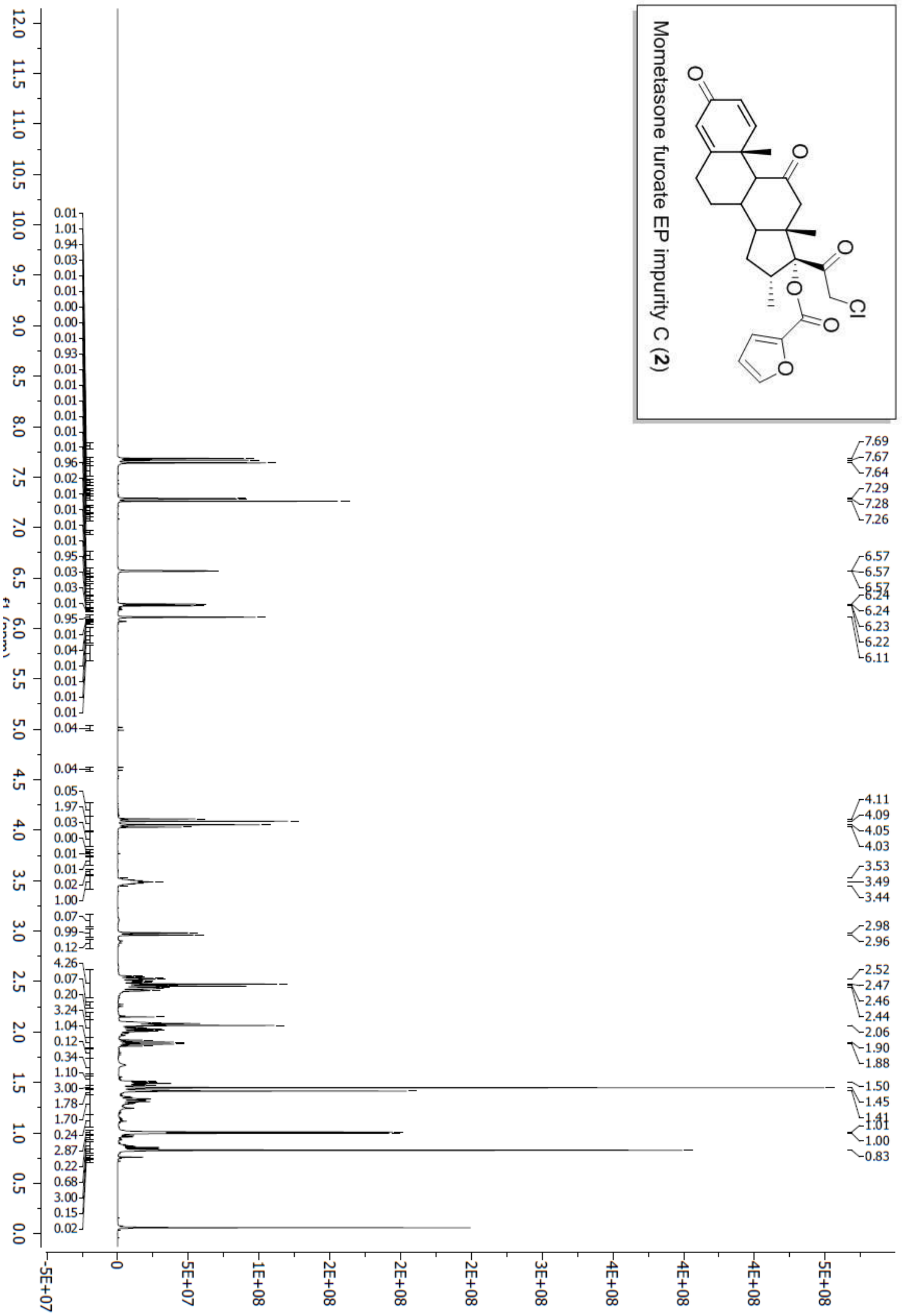

^{13}C-NMR (J-Mode, 150 MHz, $CDCl_3$) of 21'-chloro-(16'α-methyl-3',11',20'-trioxo-pregna-1',4'-dien-17'-yl) furan-2-carboxylate (mometasone furoate EP impurity C, 2)

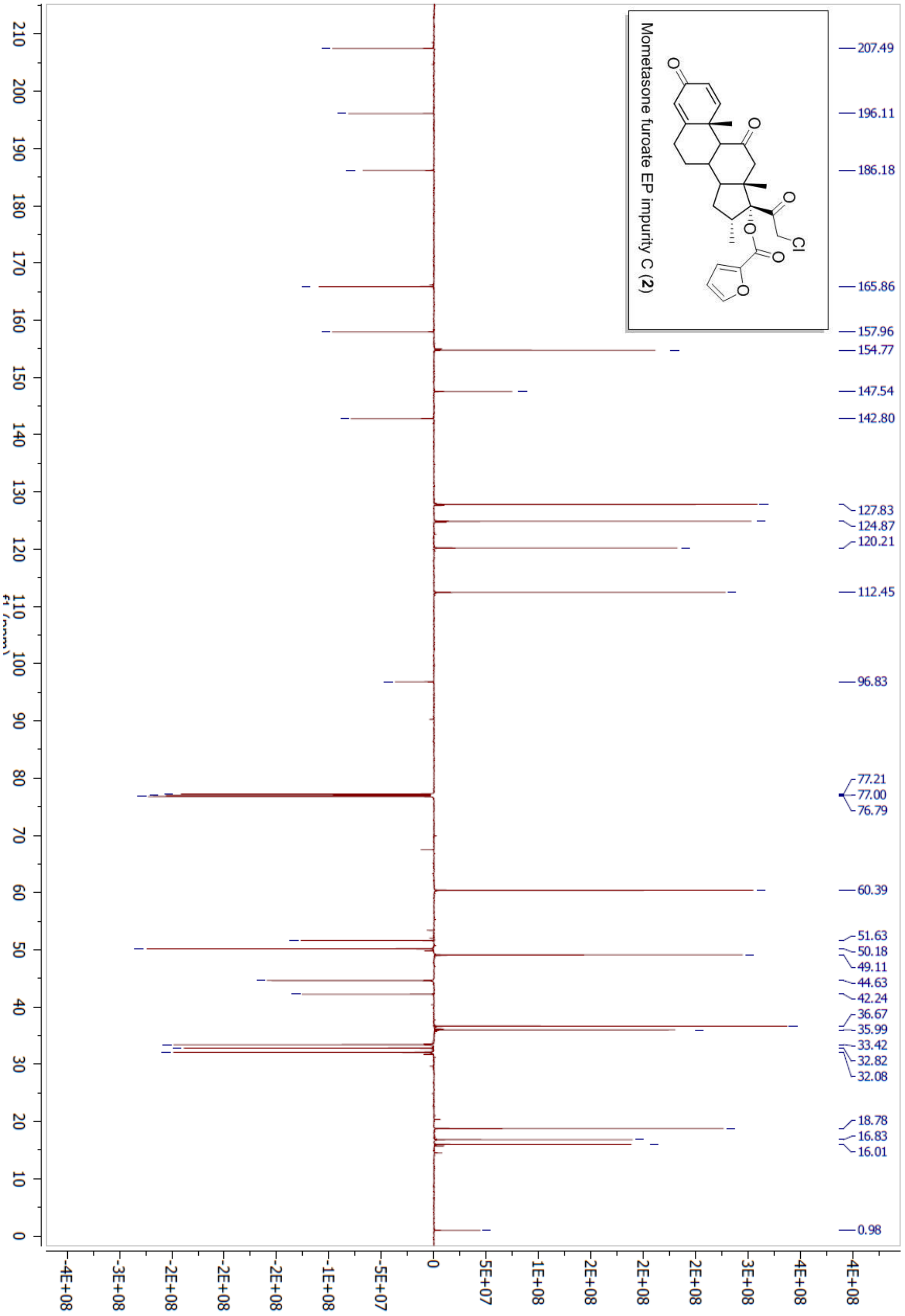

Quantitative ^{1}H-NMR (600 MHz, $CDCl_3$) of 21'-chloro-(16'α-methyl-3',11',20'-trioxo-pregna-1',4'-dien-17'-yl) furan-2-carboxylate (mometasone furoate EP impurity C, 2, 12.07 mg) in the presence of dimethylsulfone (standard for quantitative NMR, TraceCERT®) as the internal standard (1.96 mg)

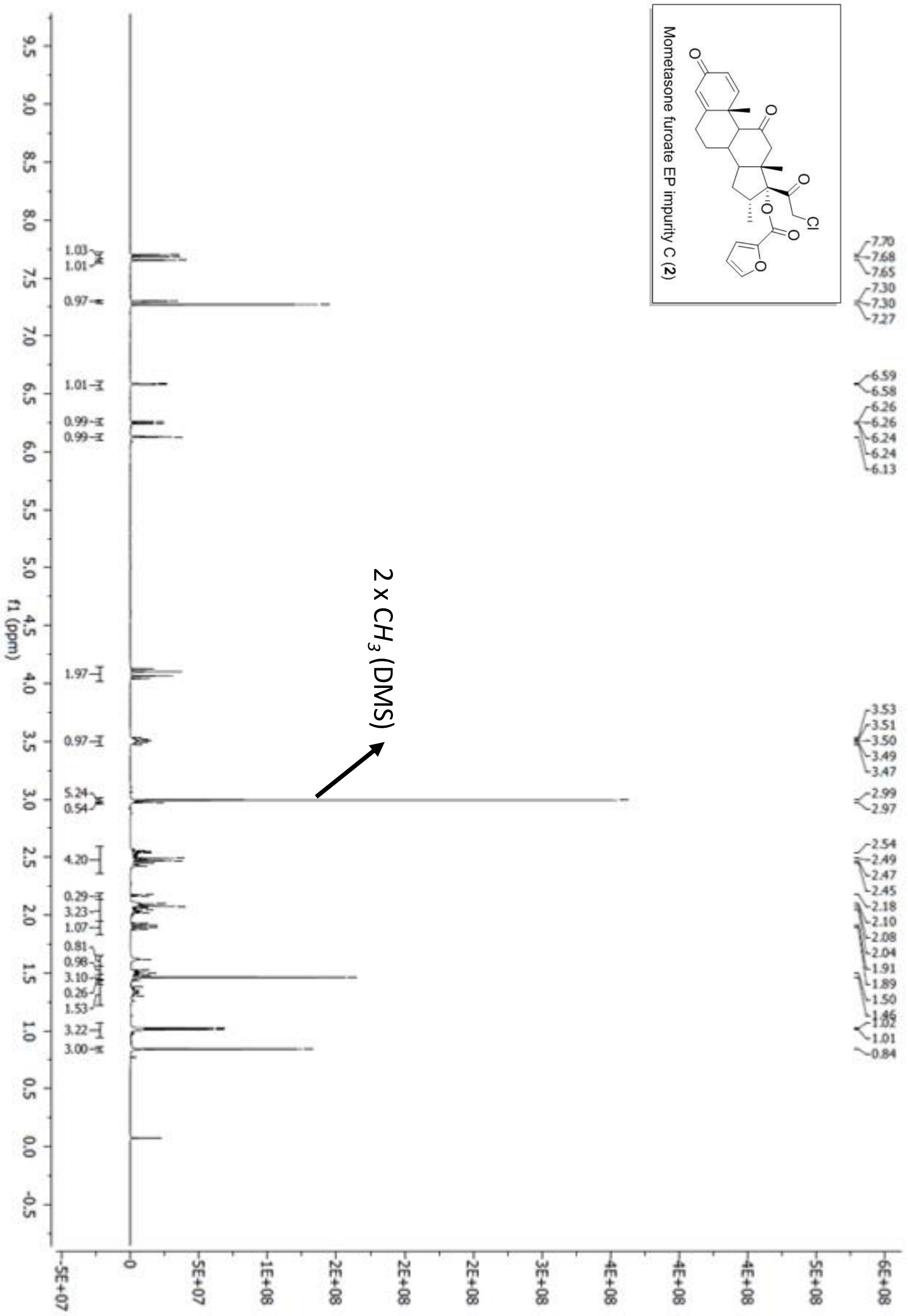

COSY (400 MHz, $CDCl_3$) of 21'-chloro-(16'α-methyl-3',11',20'-trioxo-pregna-1',4'-dien-17'-yl) furan-2-carboxylate (mometasone furoate EP impurity C, 2)

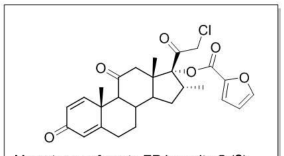

Mometasone furoate EP impurity C (**2**)

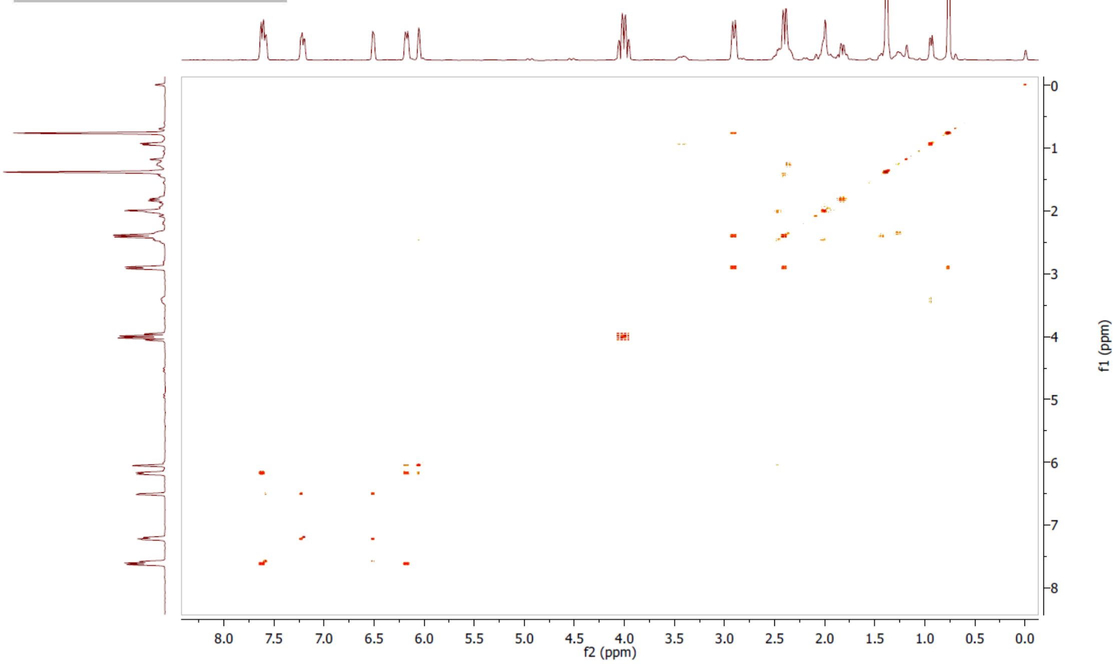

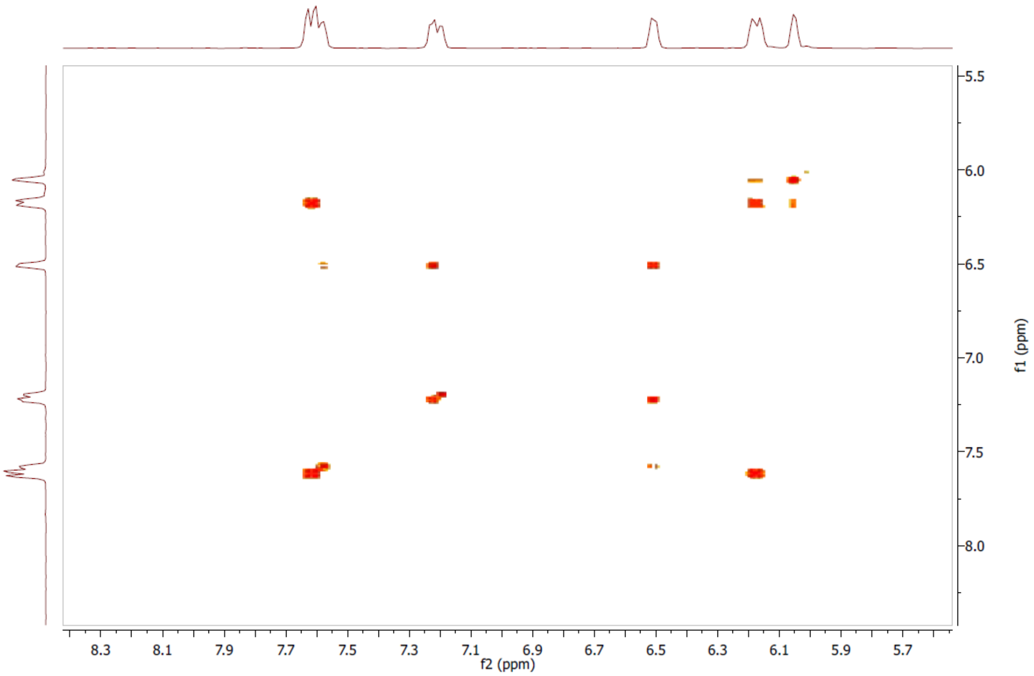

NOESY (400 MHz, $CDCl_3$) of 21’-chloro-(16’α-methyl-3’,11’,20’-trioxo-pregna-1’,4’-dien-17’-yl) furan-2-carboxylate (mometasone furoate EP impurity C, 2)

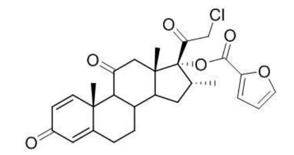

Mometasone furoate EP impurity C (**2**)

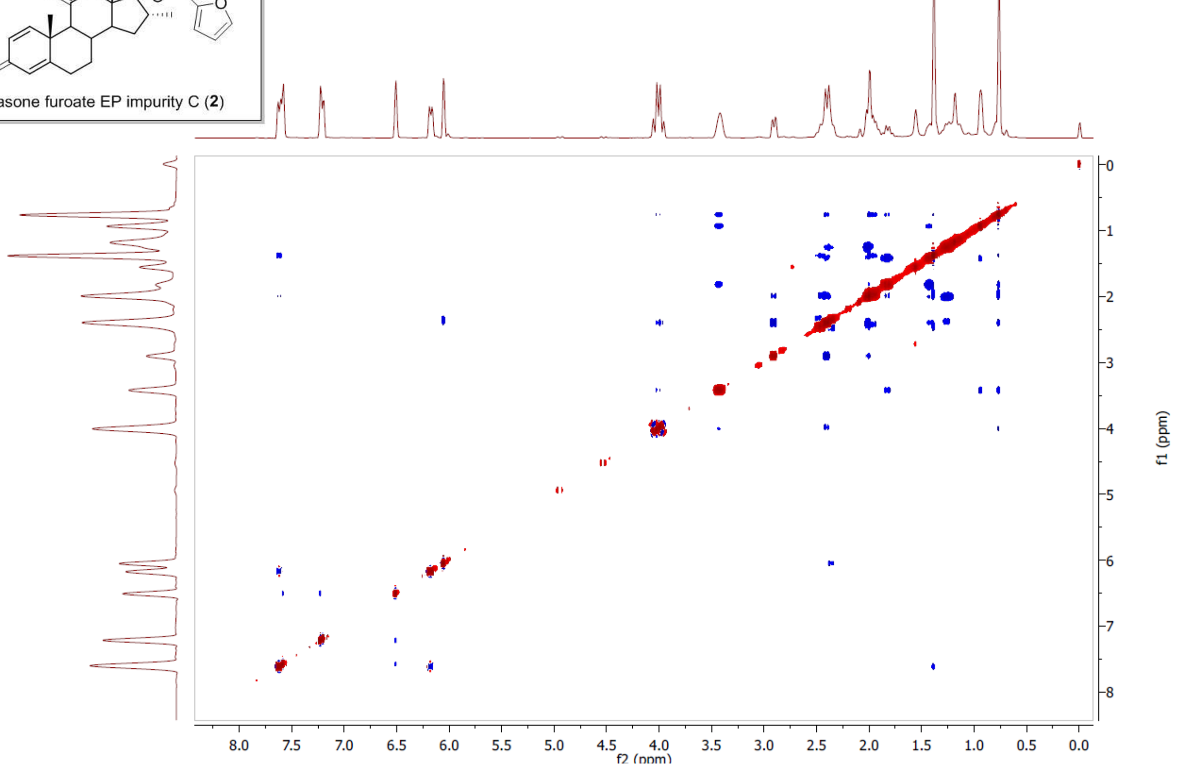

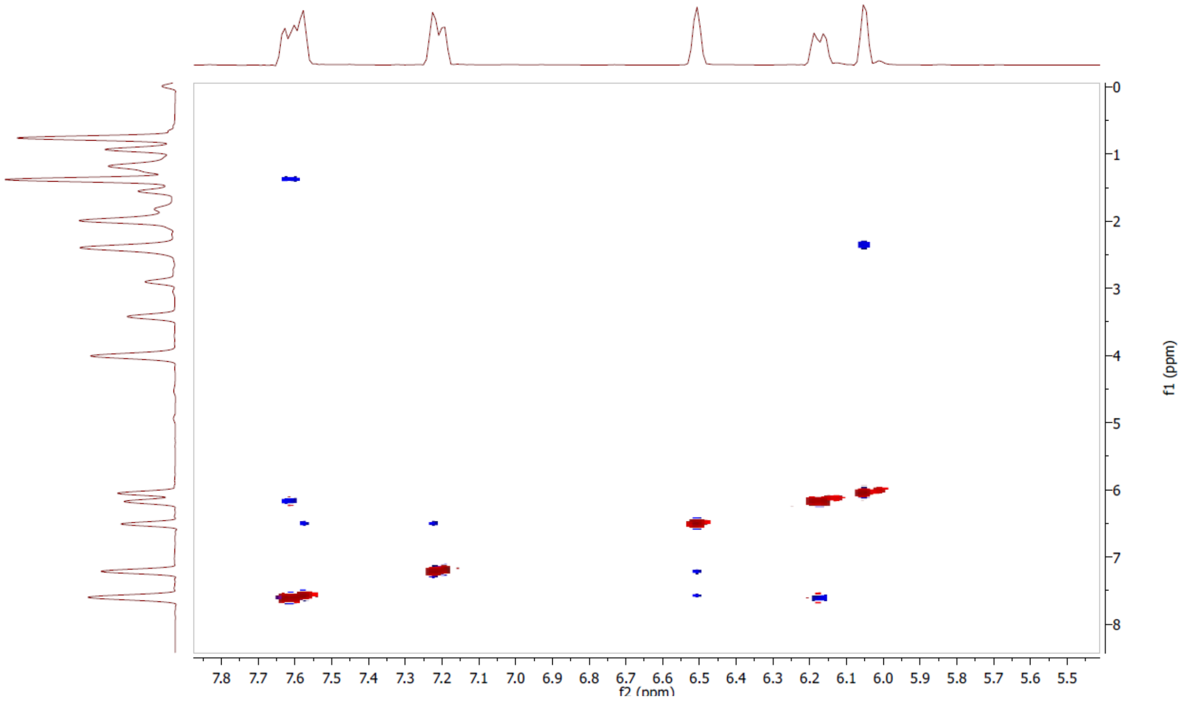

HRMS of 21’-chloro-(16’α-methyl-3’,11’,20’-trioxo-pregna-1’,4’-dien-17’-yl) furan-2-carboxylate (mometasone furoate EP impurity C, 2)

Analysis Report

Agilent Trusted Answers

Sample Information

**Name**	MomFur BC 31012023	**Data File Path**	D:\MassHunter\Data\Data\Gioiello\MomFur BC v2 31012023.d
**Sample ID**		**Acq. Time (Local)**	2/2/2023 2:33:08 PM (UTC+01:00)
**Instrument**	Instrument 1	**Method Path (Acq)**	D:\MassHunter\Methods\ACCURATE MASS DAD 31012023.m
**MS Type**	QTOF	**Version (Acq SW)**	6200 series TOF/6500 series Q-TOF B.09.00 (B9044.1 SP1)
**Inj. Vol. (ul)**	5	**IRM Status**	Success
**Position**	P1-D3	**Method Path (DA)**	D:\MassHunter\Data\Data\Gioiello\MomFur BC v2 31012023.d\Results\Qual\Version4\Default_KBr.m
**Plate Pos.**		**Target Source Path**	
**Operator**		**Result Summary**	

Sample Chromatograms

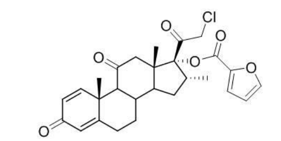

Mometasone furoate EP impurity C (**2**)

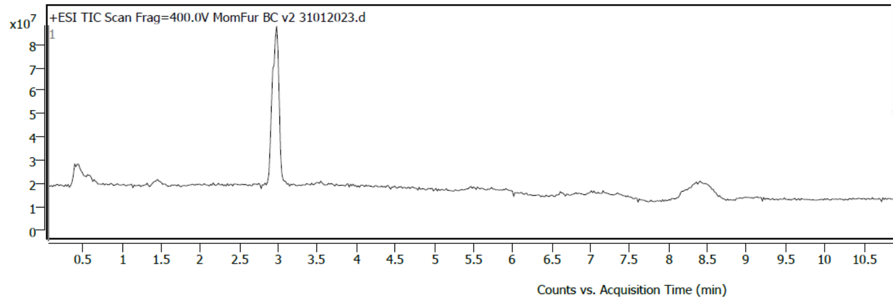

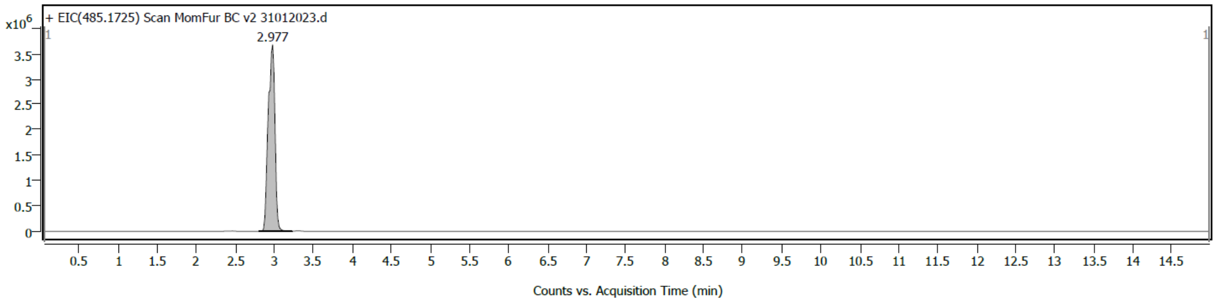

Chromatogram Peaks

Peak	Start	RT	End	Height	Area	Area %	SNR
1	2.796	2.977	3.226	3675617	22084851	100.00	

Sample Spectra

+ Scan (rt: 2.794-3.143 min) **C27 H29 Cl O6**

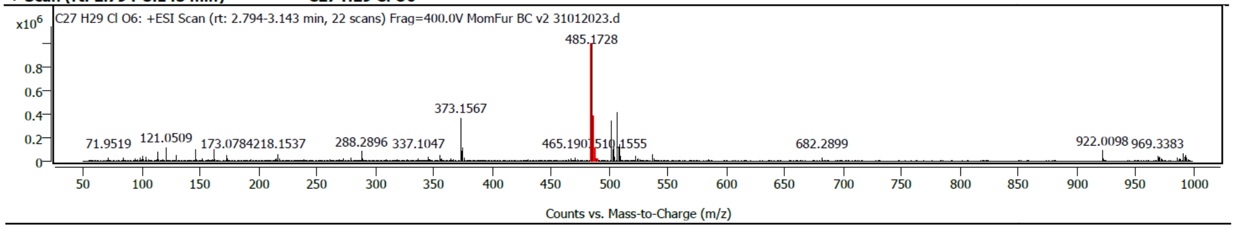

Spectrum Peaks

m/z	Z	Abund	Abund %	m/z (Calc)	Diff (ppm)	Ion Species	Formula	Ion Type
485.1728	1	1006553	100.00	485.1725	0.58	(M+H)+	C27 H29 Cl O6	
486.1763	1	274737	27.29	486.1759	0.65	(M+H)+	C27 H29 Cl O6	
487.1713	1	328904	32.68	487.1709	0.69	(M+H)+	C27 H29 Cl O6	
488.1737	1	93483	9.29	488.1736	0.27	(M+H)+	C27 H29 Cl O6	

HPLC-DAD (λ= 254 nm) of 21’-chloro-(16’α-methyl-3’,11’,20’-trioxo-pregna-1’,4’-dien-17’-yl) furan-2-carboxylate (mometasone furoate EP impurity C, 2)

Senza titolo Repor

Reported by User: System

Project Name: Impurities_Mome_beclo_antimo

SAMPLE INFORMATION

Sample Name:	mom.fvr.mix	Acquired By:	System
Sample Type:	Unknown	Date Acquired:	31/01/2023 11.14.44
Vial:	1	Acq. Method Set:	Metodo_pharmacopeia_EU_Antimo
Injection #:	1	Date Processed:	31/01/2023 15.10.13
Injection Volume:	20,00 ul	Processing Method:	Default
Run Time:	100,0 Minutes	Channel Name:	Wvln Ch1
Sample Set Name:	Impurities_antimo_31012023	Proc. Chnl. Descr.:	PDA 254,0 nm

Auto-Scaled Chromatogram

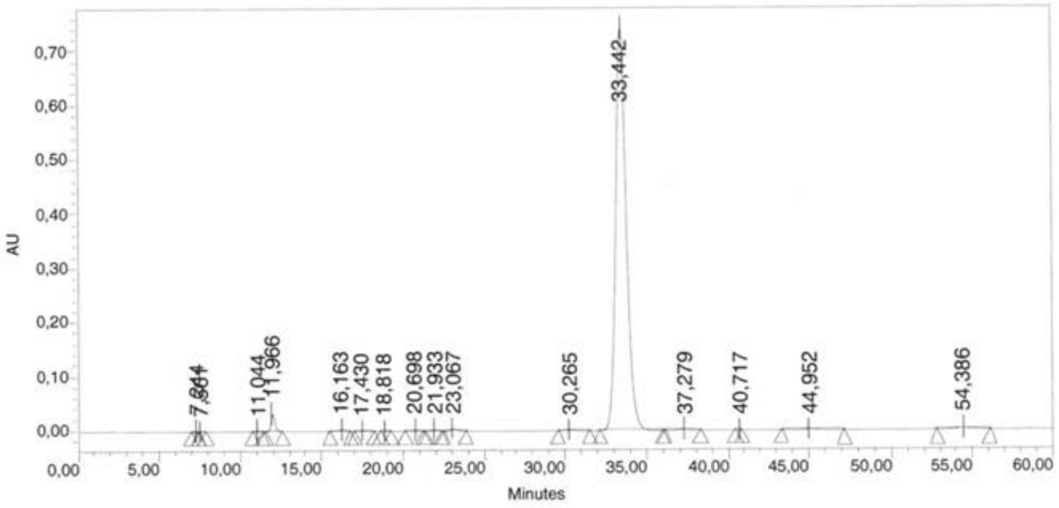

Peak Results

	Name	RT	Area	Height	% Area
1		7,244	17166	1618	0,05
2		7,561	1890	217	0,01
3		11,044	10856	708	0,03
4		11,966	545287	32113	1,56
5		16,163	76021	3144	0,22
6		17,430	23023	796	0,07
7		18,818	3256	175	0,01
8		20,698	42174	1482	0,12
9		21,933	39620	1514	0,11
10		23,067	141194	4740	0,40
11		30,265	57195	1279	0,16
12		33,442	33692423	741820	96,18
13		37,279	78351	1538	0,22
14		40,717	959	79	0,00
15		44,952	70785	829	0,20
16		54,386	229457	3187	0,66

IR spectrum of 21’-chloro-(16’α-methyl-3’,11’,20’-trioxo-pregna-1’,4’-dien-17’-yl) furan-2-carboxylate (mometasone furoate EP impurity C, 2)

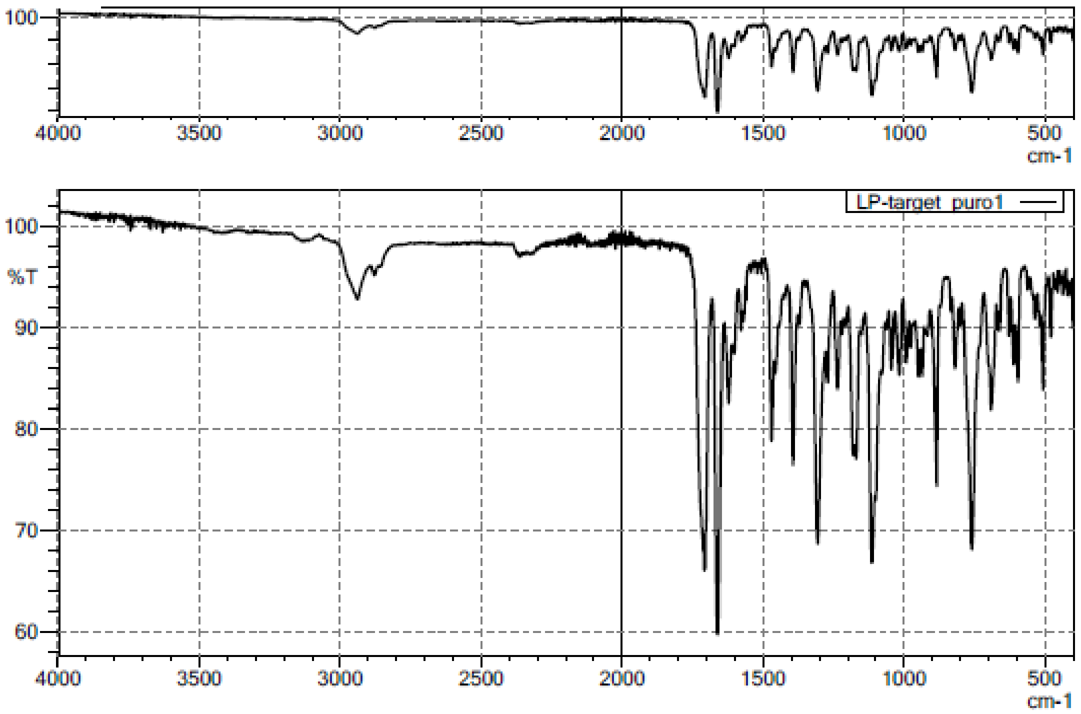

Supplement: Supplementary file 1 [file molecules-28-07859-s001.zip › molecules-2734282-supplementary.pdf]
